# Exploring the Role of Globular Domain Locations on an Intrinsically Disordered Region of p53: A Molecular Dynamics Investigation

**DOI:** 10.1021/acs.jctc.3c00971

**Published:** 2024-01-17

**Authors:** Michael J. Bakker, Henrik V. Sørensen, Marie Skepö

**Affiliations:** † Faculty of Pharmacy in Hradec Králové, Charles University, Akademika Heyrovského 1203/8, 500 05 Hradec Králové, Czech Republic; ‡ Division of Computational Chemistry, Department of Chemistry, Lund University, P.O. Box 124, SE-221 00 Lund, Sweden; § MAX IV Laboratory, Lund Institute of Advanced Neutron and X-ray Science, Scheelevägen 19, SE-223 770 Lund, Sweden; ∥ LINXS - Institute of Advanced Neutron and X-ray Science, Scheelevägen 19, SE-233 70 Lund, Sweden

## Abstract

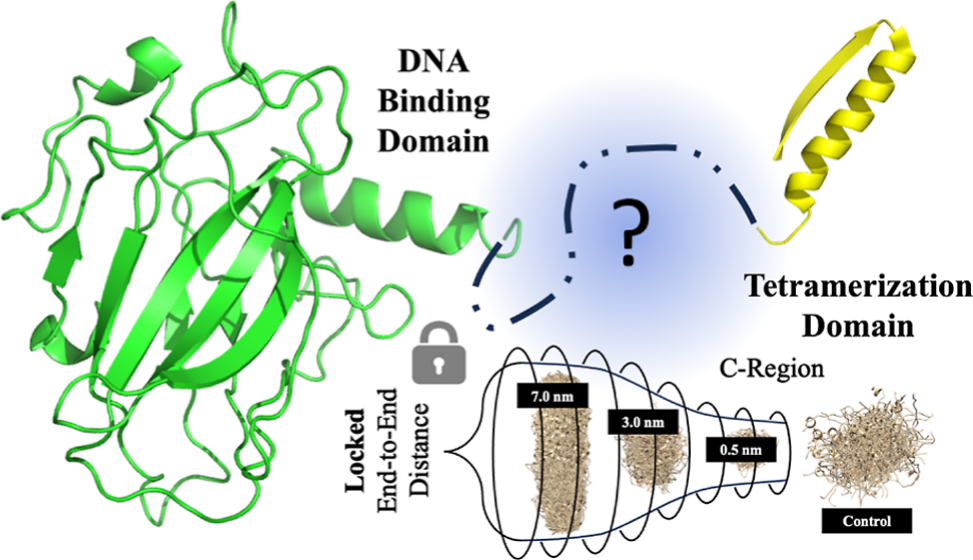

The pre-tetramerization loop (PTL) of the human tumor suppressor protein p53 is an intrinsically disordered region (IDR) necessary for the tetramerization process, and its flexibility contributes to the essential conformational changes needed. Although the IDR can be accurately simulated in the traditional manner of molecular dynamics (MD) with the end-to-end distance (EE_dist_) unhindered, we sought to explore the effects of restraining the EE_dist_ to the values predicted by electron microscopy (EM) and other distances. Simulating the PTL trajectory with a restrained EE_dist_ , we found an increased agreement of nuclear magnetic resonance (NMR) chemical shifts with experiments. Additionally, we observed a plethora of secondary structures and contacts that only appear when the trajectory is restrained. Our findings expand the understanding of the tetramerization of p53 and provide insight into how mutations could make the protein impotent. In particular, our findings demonstrate the importance of restraining the EE_dist_ in studying IDRs and how their conformations change under different conditions. Our results provide a better understanding of the PTL and the conformational dynamics of IDRs in general, which are useful for further studies regarding mutations and their effects on the activity of p53.

## Introduction

**Figure 1:**
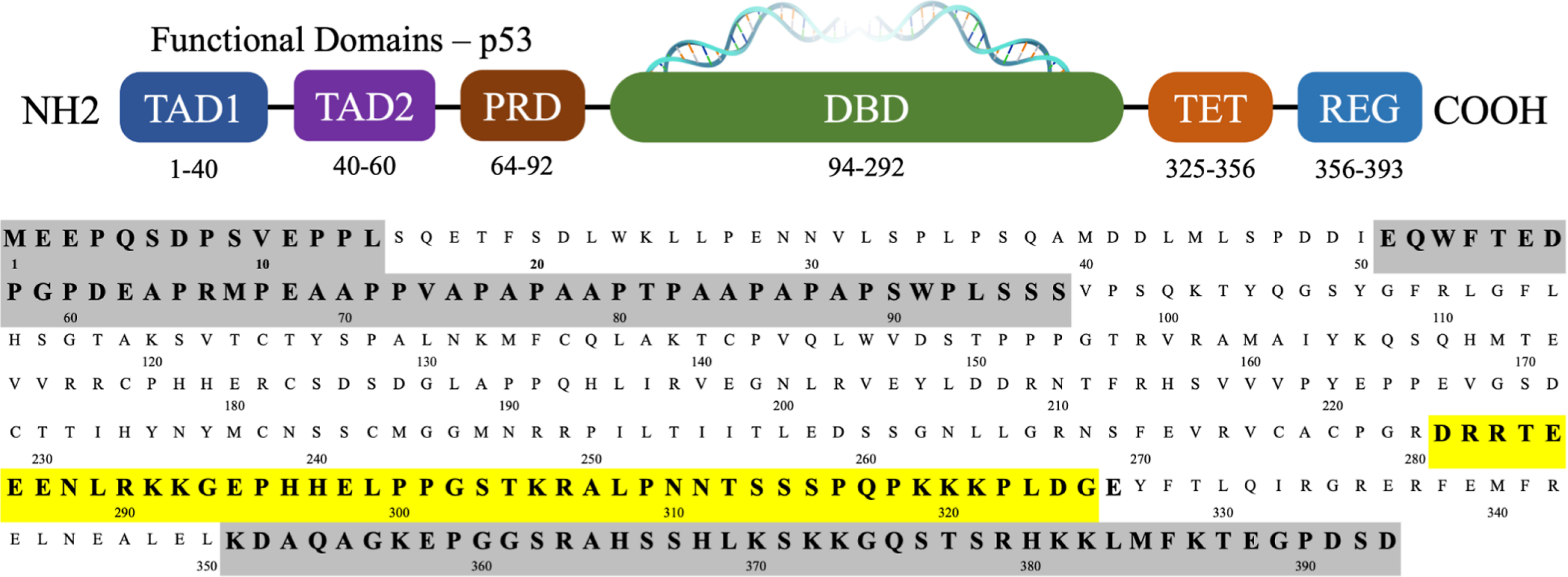
Structural analysis of the p53 functional domains and the amino acid residues presented. Disordered regions are shaded gray with the PTL highlighted in yellow.

The tumor suppressor protein p53 is vital for human cell cycle progression, playing an essential role in regulating cell division and preventing cancer development in the body.^1−4^ TP53, the gene encoding p53, is the most mutated gene in human cancers; more than 50% of cancers have mutations found within the gene.^5,6^ The p53 protein is a regulatory factor responsible for maintaining genome integrity and cell cycle control and preventing cancer development across all animal species, from humans to invertebrates.^7,8^ Research has indicated that variants and possible precursors of p53 protein have been found in organisms across the taxonomic tree.^9,10^ In its functional form, p53 binds to the DNA and searches for mutations, and upon finding them, it triggers apoptosis to prevent uncontrolled cell growth.^11^ Of major interest to the scientific community are the mutant forms of p53, as these variants have been observed to possess oncogenic characteristics.^12,13^ p53 is 393 amino acids long and contains several functional domains and four IDRs, all of which are depicted below (Figure 1).

The p53 protein contains a DNA binding domain (DBD),^1,2,14−16^ a transactivation domain (TAD) divided into two regions responsible for activating transcription,^2,17^ a proline-rich domain (PRD),^3,18^ a regulatory domain (REG) containing phosphorylation sites to activate and deactivate the protein,^2,19,20^ and the tetramerization domain (TET) which enables the oligomerization of the p53 monomers.^1,2,21^ The TET contains an α-helix and β-sheet which enable the p53 monomers to link in tandem.^22^ Crucially, it also contains the pre-tetramerization loop (PTL) between residues Lys_292_ and Gly_325_, an intrinsically disordered region that allows the p53 monomers to link, increasing the DNA binding affinity 1000-fold.^23^ This region provides the flexibility necessary for tetramerization; however, the exact mechanisms of this process and the specific conformations of the PTL pre/post-tetramerization are unknown.

**Figure 2:**
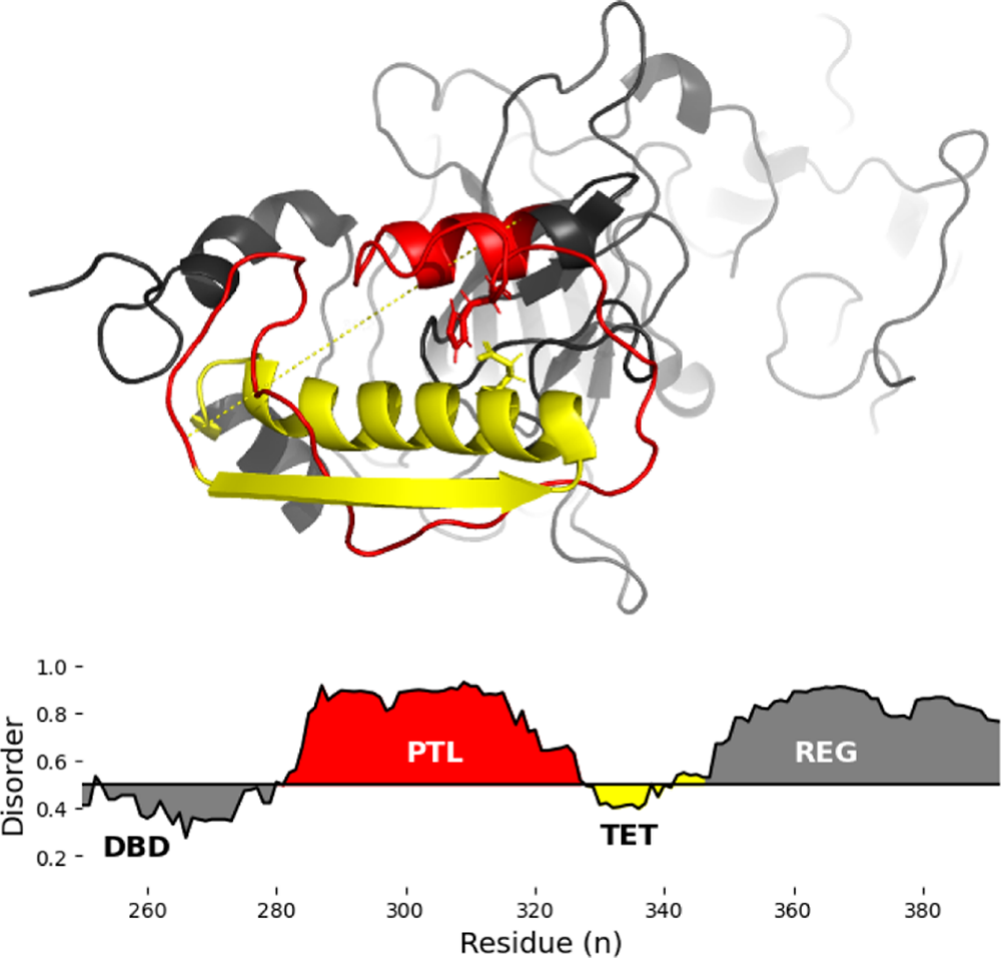
3D representation derived by electron microscopy^24^ (top) for the monomeric form of this region with the TET region highlighted in yellow and the PTL in red and the predicted disorder (bottom) for the p53 protein displaying the intrinsically disordered PTL and REG regions connected by the TET domain based on IUPRED.^25^

While much of the p53 is well studied, the PTL region and other IDRs are often ignored due to their challenging nature, as traditional biophysical and structural biology methods used to characterize globular proteins have limited use for intrinsically disordered proteins (IDPs) and IDRs.^26−28^ This makes determining protein–protein interaction sites, structural dynamics, and conformational analysis difficult.^27,29^ Fortunately, advances in computational techniques, such as molecular dynamics (MD) with force fields^30,31^ specifically designed to incorporate the inherent disorder, provide researchers with great insight into the inner workings of these regions.^28^ IDRs are typically involved in regulatory functions and require as much flexibility as possible.^32^ This might explain why IDRs are commonly found in proteins at the N- or C-terminal and less frequently internally.^33^ However, flexibility is also important for conformational changes in the protein, such as the oligomerization processes.^16,34,35^ The PTL region is such a sequence in p53, as noted from the degree of disorder (Figure 2). The PTL segments contain 31 amino acids generally believed to be disorder-promoting (Ala, Arg, Gly, Gln, Ser, Glu, Lys, and Pro) based on sequence analysis of IDRs and IDPs.^33^ In contrast, PTL only contains seven amino acids that are believed to be order-promoting, four Leu and three Asn (the residues believed to promote order are Trp, Tyr, Phe, Ile, Leu, Val, Cys, and Asn).^36^

Terminal IDRs are found at the periphery of the sequences^33^ and their movements and dynamics are minimally restricted by being attached to only one globular domain. For internal IDRs, however, both ends are attached to the globular domains. These flexible linkers and spacers can allow domains to sample a wide range of positions with respect to one another and act as flexible loops with solvent-accessible surface areas that can interact with the globular regions. However, being attached to two structural domains limits the number of conformations the IDR can adopt and restricts the region’s dynamics. The increased mass at the end of the IDR hinders movement, and some conformations are forbidden due to the steric hindrance of the globular domains. In the case of some IDRs, including PTL, the structural domains have large interaction partners that lock the protein’s domains in place and either severely slow or even arrest the movement of the domains with respect to each other. This locks the IDR’s EE_dist_ which restricts the conformational ensemble to only include conformations within a specific end-to-end span. The effect of such situations on the conformational ensembles remains largely unexplored.

In IDRs, it is often observed that specific conformations permit the existence of small transient secondary structures that facilitate the conformational changes in the protein.^37^ Understanding the influence of end-to-end span on the IDR’s structure, behavior, and interactions is therefore vital to uncovering the impact of conformational changes in the protein on secondary structure in IDRs, and vice versa. By studying the PTL with MD simulations as a free IDP and as an IDR with different restrained EE_dist_ , we see how the restriction on the movement of the terminals of the IDR affects the conformations it can take and what secondary structure it can assume. We compare the data with previously obtained electron microscopy structure^24^ of the p53 monomeric form (PDB: 8f2i) to understand how the region settles when the protein is inactive. The local interactions were evaluated by comparing predicted chemical shifts (CSs) to solution NMR data^38^ to assess the fidelity of the models. Through this research, it is hoped that a better understanding of the influence of the end-to-end span of residues on the structure, behavior, and interactions of the region can be gained.

## Materials and Methods

Atomistic MD simulations were performed using the GROMACS package version 2022.^39−42^ The AMBERSB99-ILDN force field^43^ and 4-point TIP4P-D water model^44^ were chosen based on similar investigations for their capacity to simulate IDPs at prolonged lengths without a bias toward secondary structure.^45−49^ The starting structure for the unrestrained trajectory was generated using Avogadro,^50^ thereafter allowing the structures to relax. The termini were simulated in their zwitterionic state, and the charges of the side chains were set to physiological pH, giving the PTL a net charge of +3. The zwitterionic state was chosen to replicate a number of investigations into similar internal regions of IDRs. In the future, investigations into the influence of the charged termini would be fruitful, although for this investigation, this variable was not tested. In terms of NMR, however, if there is an influence on the CSs, then it is most likely localized, particularly in trajectories whose end termini are positionally restrained. The periodic boundary conditions were simulated with a rhombic box with a minimum distance of ten nm from the PTL residues, in all directions. All trajectories were solvated with ions sufficiently to neutralize the charged residues only (minimum of three chlorine atoms).

A “pool” of several thousand randomized structures was generated using Flexible-Meccano^51^ to simulate hindered termini trajectories and then sorted by their EE_dist_. One structure was chosen (EE_dist_ = 0.5 nm) to represent the fully contracted state, and another structure was chosen (EE_dist_ = 7.0 nm) for the expanded state to understand the region under extreme conditions. Two other structures were chosen, one at EE_dist_ = 3.0 nm to reflect the electron microscope structure and SAXS rigid body model prediction in the monomeric form (3.02/3.27 nm), and one at EE_dist_ = 5.0 nm as seen in the EM/SAXS structure when p53 is DNA bound (4.10/5.33 nm).

Each simulation was implemented using the leapfrog integrator with a time step of two fs. Neighbor searching was conducted through the Verlet scheme by a grid algorithm using a cutoff of 12 Å, and the electrostatic potential was implemented through the particle mesh Ewald (PME) method. Temperature coupling was achieved by the Parrinello–Rahman barostat, and the Nose–Hoover thermostat was used to maintain a temperature of 298 K. The LINCS algorithm was employed with hydrogen bond constraints. The simulations were minimized by using the steepest descent algorithm. The system was then equilibrated at constant pressure (NVT) for 500 ps and at constant volume (NPT) for one ns. An additional 100 ns of relaxation time was given to ensure the system was not oversampling high-energy states before trajectory collection began. Each trajectory was then given time to explore its corresponding free energy landscape, generating frames at each ten ps for analysis. For the restrained trajectories, an additional command was included in the MDP file, *freeze_grps*, to lock the *x*,*y*,*z* positions of the start and end residues (Glu_281_ and Gly_325_). The trajectories were simulated in replicates of one μs, as seen in Table 1 with total varying lengths of five μs for the unrestrained trajectory, and four μs for each of the restrained trajectories. The only exception is EE_dist_ = 7.0 nm, as it was quickly deemed to contain very little conformational variation.

**Table 1:** Simulation Parameters for the PTL Trajectories^a^

Traj.	*E*_dist_ (nm)	Cl^–^	Na^+^	time
nCp53		3	0	1 + 1 + 1 + 1 + 1
aCp53	0.5	4	1	1 + 1 + 1 + 1
bCp53	3.0	4	1	1 + 1 + 1 + 1
cCp53	5.0	7	4	1 + 1 + 1 + 1
dCp53	7.0	12	9	1 + 1

a The restrained trajectories (*E*_dist_) are given in nanometers, the number of Cl^–^ and Na^+^ ions, and the total simulation time (in μs).

Analysis of the simulations was performed using a variety of tools and packages. We computed the autocorrelation of the radius of gyration (*R*_g_) of each atom in the molecule for each time step of the MD trajectory (see the Supporting Information). We used this autocorrelation analysis to observe how the radius of gyration changes over time in the system. The *R*_g_ was computed using the MDTraj Python packages.^52^ Theoretical scattering intensities were generated by CRYSOL, version 3.0.3.^53^ The scattering intensities were investigated in order to provide a visualization of the conformational properties of our system, even when not directly compared to the experimental SAXS data. Secondary structures were determined by the DSSP-PPII program, which permitted the determination of left-handed polyproline II and other traditional secondary structures defined by DSSP.^54^ NMR Chemical shift predictions were generated by the neural-network-trained Sparta+ suite of codes.^55^ Free energy plots were generated using the PyEMMA python scripts^56^ and the Campos and Baptista approach,^57^ and dimensionality reduction (DR) such a principle component analysis (PCA) and clustering (K-means) were done by the python packages included in SciPy/Sklearn.^58^ PRIMUS was used to generate pairwise distribution plots,^59^ and PyMol^60^ and Chimera^61^ were utilized for visualizations of the protein.

## Results and Discussion

### p53 PTL Treated as an IDP

**Figure 3:**
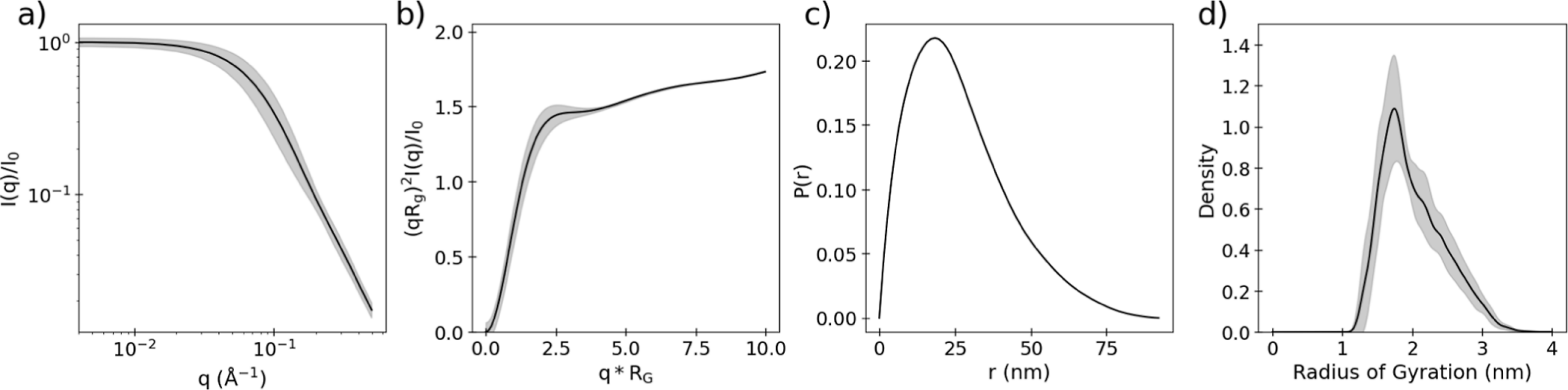
Form factor (a) comparing the scattering vector (q) to the normalized intensity (I(q)/I(0), dimensionless Kratky plot (b) incorporating the *R*_g_, pairwise distance distribution (c) of the distances between atoms (r) and the probability (P(r)) that they will be found in such state, and *R*_g_ distribution (d) for the 5 μs unrestrained trajectory. Shaded regions represent the standard deviation between each replicate.

A structural ensemble was generated at a time step of ten ps from the PTL unrestrained trajectory using the gmx *trjconv* tool. The theoretical scattering curves from the CRYSOL predictions of the simulation show a strongly disordered region (Figure 3a). The dimensionless Kratky plot (Figure 3b) indicates that the trajectory, in its unhindered state, is highly flexible and disordered. The pair distance distribution function (Figure 3c) is a statistical measure used to analyze the distribution of distances between pairs of atoms within a system. The distances for the unrestrained PTL region are distributed from 0 to 80 nm, with a singular peak value of around 25 nm. The shape of the curve suggests a disordered structure that is not trapped in specific conformational wells, sampling a sufficient amount of the phase space available. The *R*_g_ plot (Figure 3d) suggests that the trajectory settles comfortably at about 1.8 nm. However, it can expand up to 3.4 nm and naturally contract to around one nm in its unrestrained form. Additionally, the trajectory was assessed using autocorrelation calculations, as seen in the Supporting Information.

**Figure 4:**
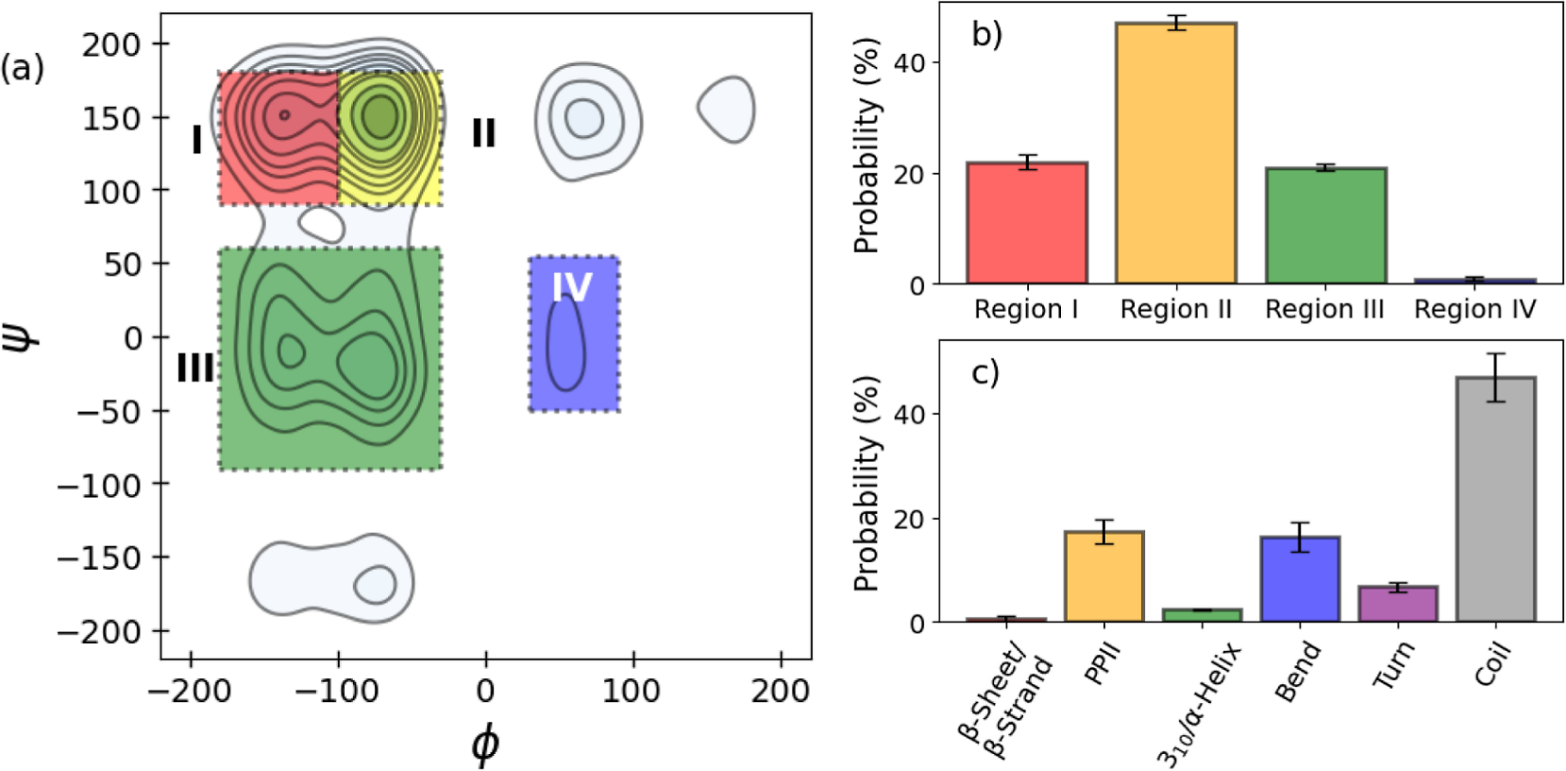
Secondary structure predictions based on the distribution of phi and psi angles in the integrated Ramachandran plot (a). Four distinct regions were highlighted for their propensity (b) to form specific secondary structures; (I) β-strands, (II) poly proline type II helices, (III) 3_10_- and right-handed α-helices, and (IV) left-handed α-helices. Propensity is also shown using DSSP-PPII (c) for comparison.

By compiling the dihedral angles into an “integrated Ramachandran plot,” we understand the possible secondary structures sampled in the trajectory. The distribution of dihedral angles is expressed (Figure 4a), with four regions of interest, as described in a similar investigation.^62^ These regions are commonly associated with the formation of specific secondary structures, including β-strands (I), polyproline type II (PPII) helices (II), 3_10_- and right-handed α-helices (III), and left-handed α-helices (IV). The relative distribution of these dihedrals in the unrestrained trajectory shows the prospective secondary structures available (Figure 4b). The majority of the trajectory is purportedly in the PPII region (∼45%) with very little to no (<3%) left-handed α-helices. In addition to the dihedral angles, a DSSP-PPII analysis of the MD simulations was performed to obtain an estimated secondary structure (Figure 4c).

From this analysis, most of the trajectory seems to be unstructured, with some instances of helices, bends, and turns. While the integrated Ramachandran suggests the presence of β-strands or α-helices that are not detected in such quantities in the DSSP-PPII predictions, there is agreement for PPII helices. The integrated Ramachandran plot shows that ∼45% of the replicates exist in a region associated with PPII helices, and the DSSP-PPII analysis detected ∼19% of the unrestrained trajectory present structures that have been identified as such. This is expected, as PPII helices have been observed frequently in disordered regions of proteins. As opposed to the common helical structures, the PPII helices have little to no hydrogen bonding capacity and have been observed to play a role in interactions between the domains of the proteins.

**Figure 5:**
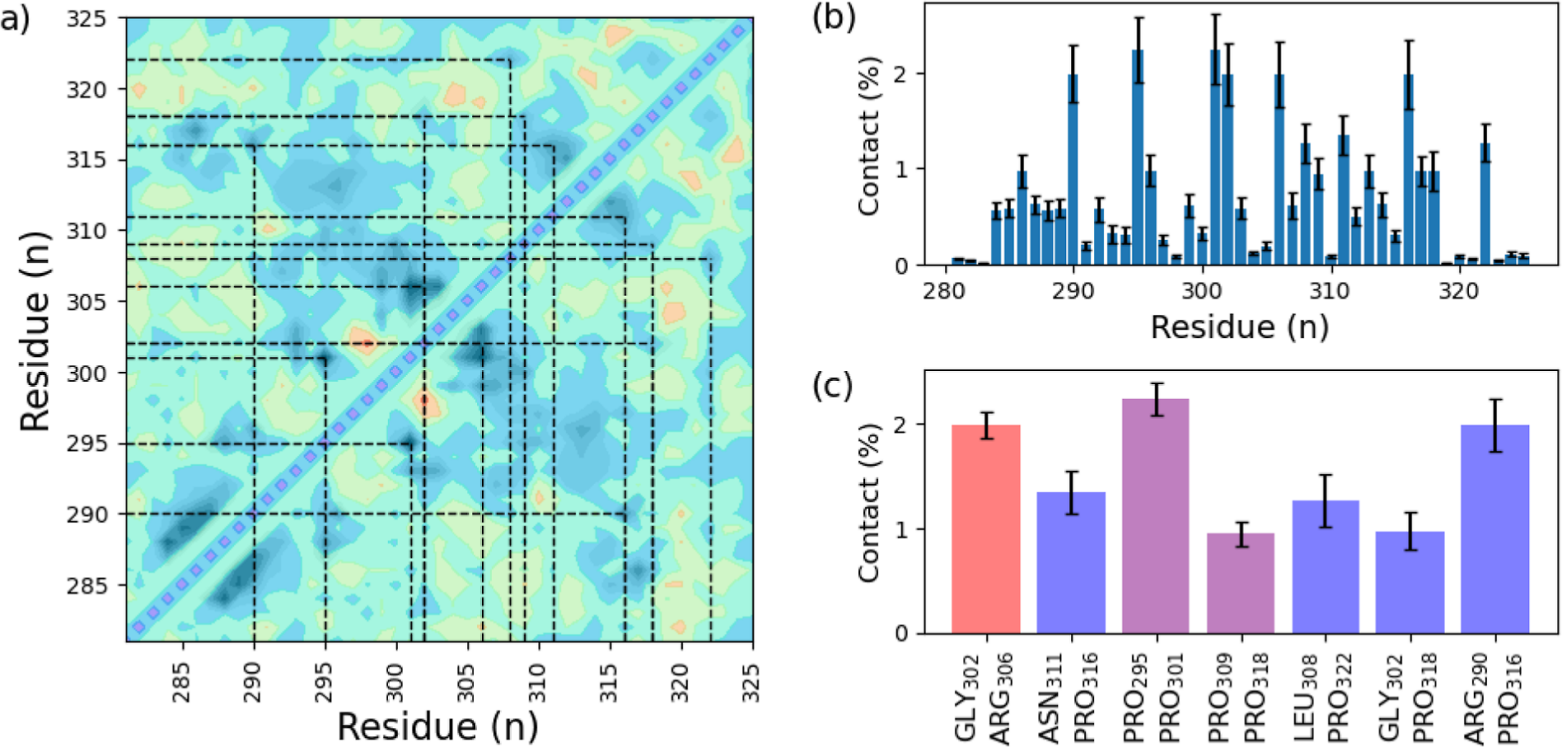
Minimum distance map (a) of the unrestrained trajectory determined between each residue (colored) as well as the probability of contact (within 0.3 nm) between each residue shaded black overlaid upon each other. Sites of notable and regular contact are highlighted with dotted lines. The maximum number of contacts is projected (b), and the seven most notable contacts are plotted (c) with shading according to the presence of proline. Purple bars are proline–proline interactions; red bars contain no proline interactions; and blue bars are proline–*x* residue interactions.

In addition to the transient secondary structure in the largely disordered regions, the intramolecular contact between the residues is of note for loop dynamics. The minimum distances observed between any given residue pair were plotted for analysis (Figure 5). Regions shaded in blue or purple represent residues that are permitted to contact. By contrast, regions shaded orange or red are residues that are not capable of interacting. These regions are significant because, in regions where the residues are in contact and interacting (e.g., hydrogen bonding), there are typically adjacent regions that make contact conformationally unobtainable or extremely unlikely. Figure 5a is also overlaid with a second black/white contour plot, demonstrating the probability that the two residues are in contact as a significant portion of the total trajectories. Dotted lines are drawn between the residues to discern these high-probability interaction sites.

Several residues share significant contact in the unrestrained trajectory (Figure 5a). The maximum number of contacts per residue is plotted (Figure 5b), with several contacts spread across multiple residues. In contrast, others are concentrated between specific points (Figure 5c). Nearly all of these residue interactions occur between residue pairs containing either one (blue) or two (purple) proline residues. Only one significant site of interaction was observed between nonproline residues, GLY_302_ and ARG_306_ (red), highlighting the oftentimes overlooked importance of the proline residues’ contribution to the overall structure in IDPs/IDRs.

### p53 TET Treated as an IDR

**Figure 6:**
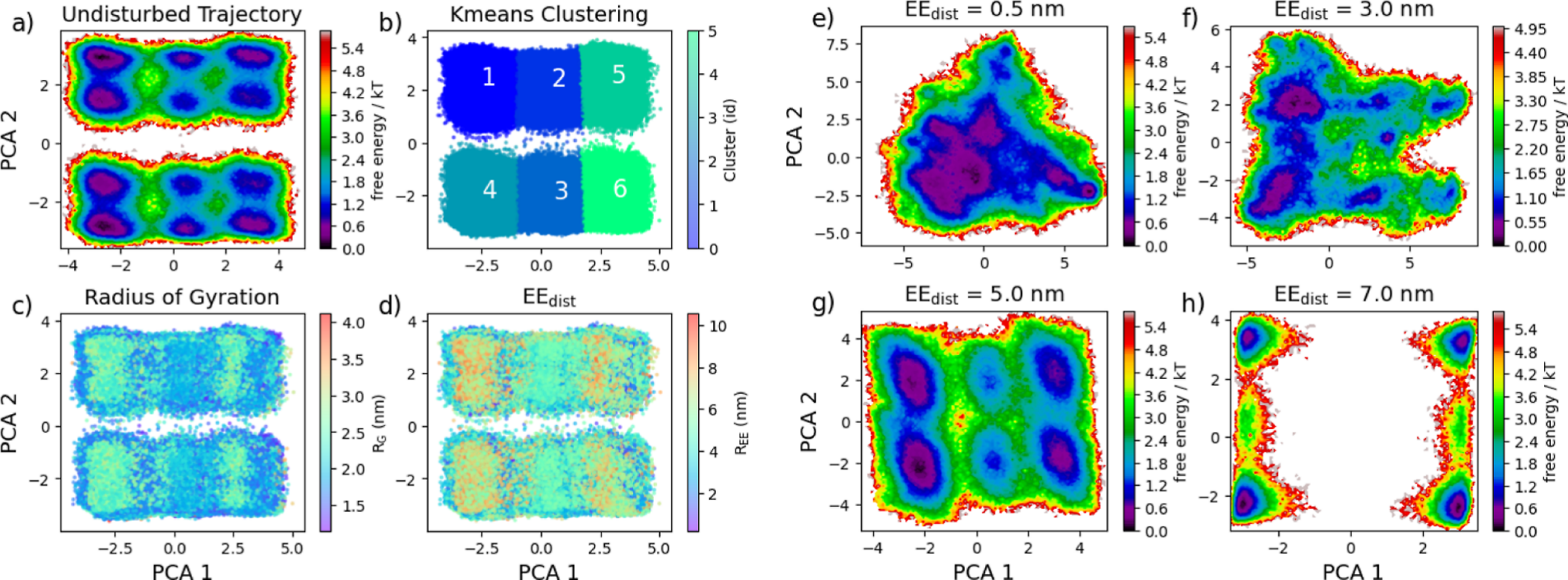
Dimensionally reduced free energy surfaces of the unrestrained trajectory (a) as well as the restrained trajectories (e–h). Different regions of the unrestrained trajectory were clustered (b) using K-means clustering and evaluated for the individual frames’ *R*_g_ (c) and EE_dist_ (d).

So far, analysis has been concentrated on the PTL region simulated as an independent free-moving IDP. We now seek to compare with the terminally restrained trajectories to gain an understanding of the IDR as its energy landscape diverges. The shape factor analysis for the restrained trajectories can be seen in the Supporting Information, although since the trajectories were artificially restrained, the results are negligible. DR techniques such as PCA allow us to compress large data sets, identifying meaningful patterns. Figure 6a shows a PCA DR on the ϕ and ψ dihedrals in the unrestrained trajectory, with the free energy estimated using PyEMMA’s *plot_free_energy* tool.^56^ The reduction splits the trajectory into six distinct clusters clustered using *k-means* clustering (Figure 6b). Evaluating the clusters by *R*_g_ (Figure 6c) and EE_dist_ (Figure 6d) we see that the clusters follow specific patterns corresponding to collective variables. Clust_1_ and Clust_3_ seem to have high EE_dist_ and *R*_g_, while clusters Clust_5_ and Clust_6_ are relatively low for both. It appears that clusters Clust_2_ and Clust_4_ contain a mixture of both high and low values.

Applying this machine learning technique to the restrained trajectories shows that the conformational landscape is greatly altered by restricting the end terminals (Figure 6e–h). Comparing these landscapes, we can interpret a bifurcation in the available conformations present when the end terminals are hindered by globular regions, as in internal IDRs. In the contracted state (EE_dist_ = 0.5 nm), the trajectory exhibits a significant region of the phase space at lower energy states, with few to no restrictions on the conformations sampled. As the trajectory is simulated at a more expanded state (EE_dist_ = 3.0 nm), we see the development of restricted regions and energetically unfavorable states. At an EE_dist_ of 5.0 nm (Figure 6g), the landscape begins to resemble the unrestrained PTL trajectory, with noticeably reduced regions akin to Clust_5_/Clust_6_ (Figure 6b). When the simulation is extended (EE_dist_ = 7.0 nm), the landscape presents strong separations between the conformations and rarely settles in energetically favorable conformations (Figure 6h).

**Figure 7:**
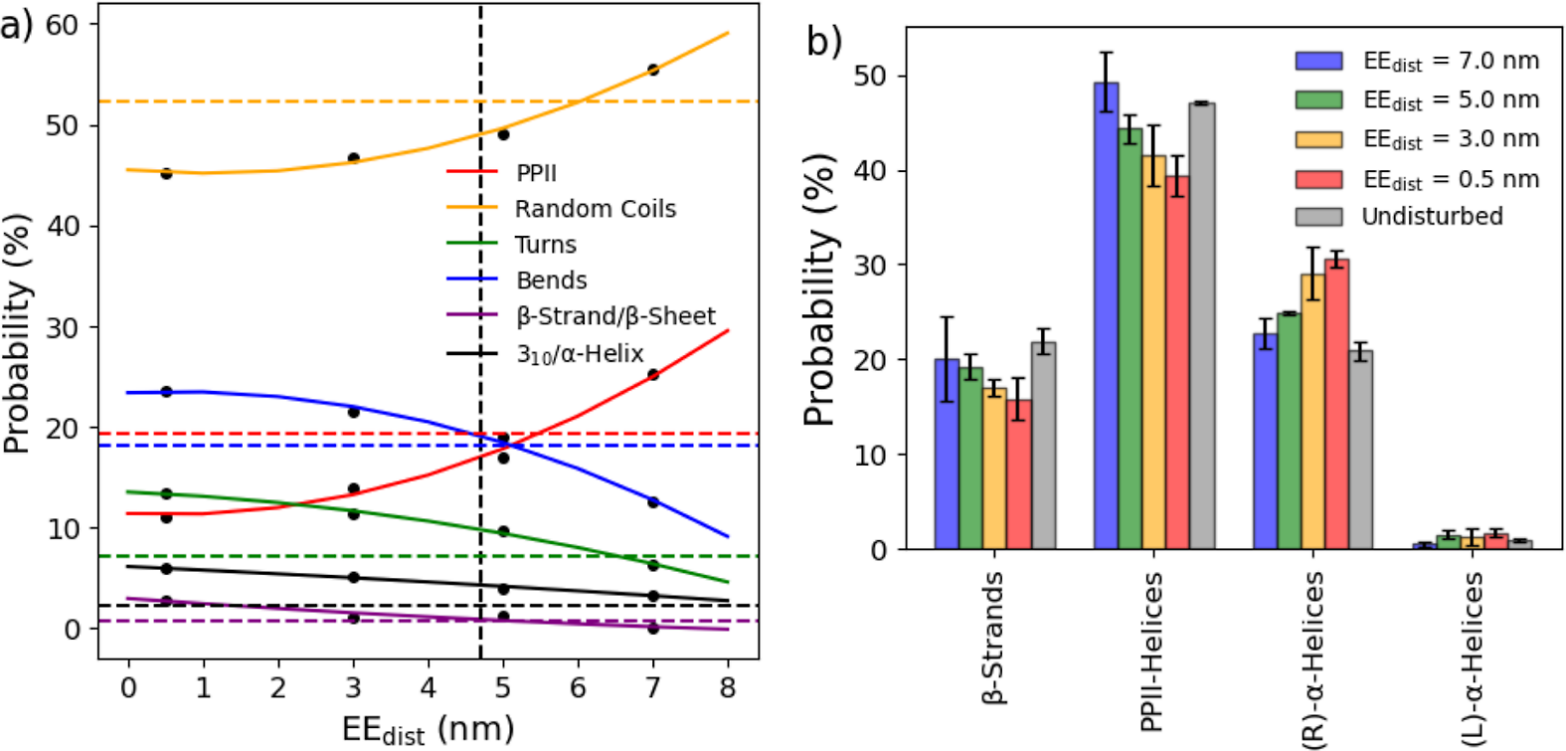
Relative distribution of polyproline type II helices, random coils, turns, bends, β-strands and β-sheets, and α-helices (a) at different EE_dist_ values predicted by DSSP-PPII (solid lines). The average EE_dist_ (vertical) and the distribution of the secondary structure from the unrestrained trajectory (horizontal) are plotted with dotted lines. The distribution of dihedral angles in specific regions of the Ramachandran plot (b).

Evaluating the observed secondary structure (Figure 7a), several notable trends emerged. As the EE_dist_ increases, random coils are increased, while all other instances of secondary structure (bends, turns, α-helices, and β-sheets/strands) decrease. The one exception to this trend is the PPII helices, which noticeably increase from ∼10% at 0.5 nm to ∼21% at 7.0 nm. The integrated Ramachandran plot (Figure 7b) also shows a similar trend, with the regions associated with PPII helices increasing upon expansion, while those about α-helices decrease. This interchange in structure between more globular-related moieties to intrinsically disordered structures upon expansion is a significant clue to interpret how the p53 tetramerization process is implemented. In simpler terms, stabilization at extended states appears to depend more on the formation of PPII helices than those that stabilize globular regions.

**Figure 8:**
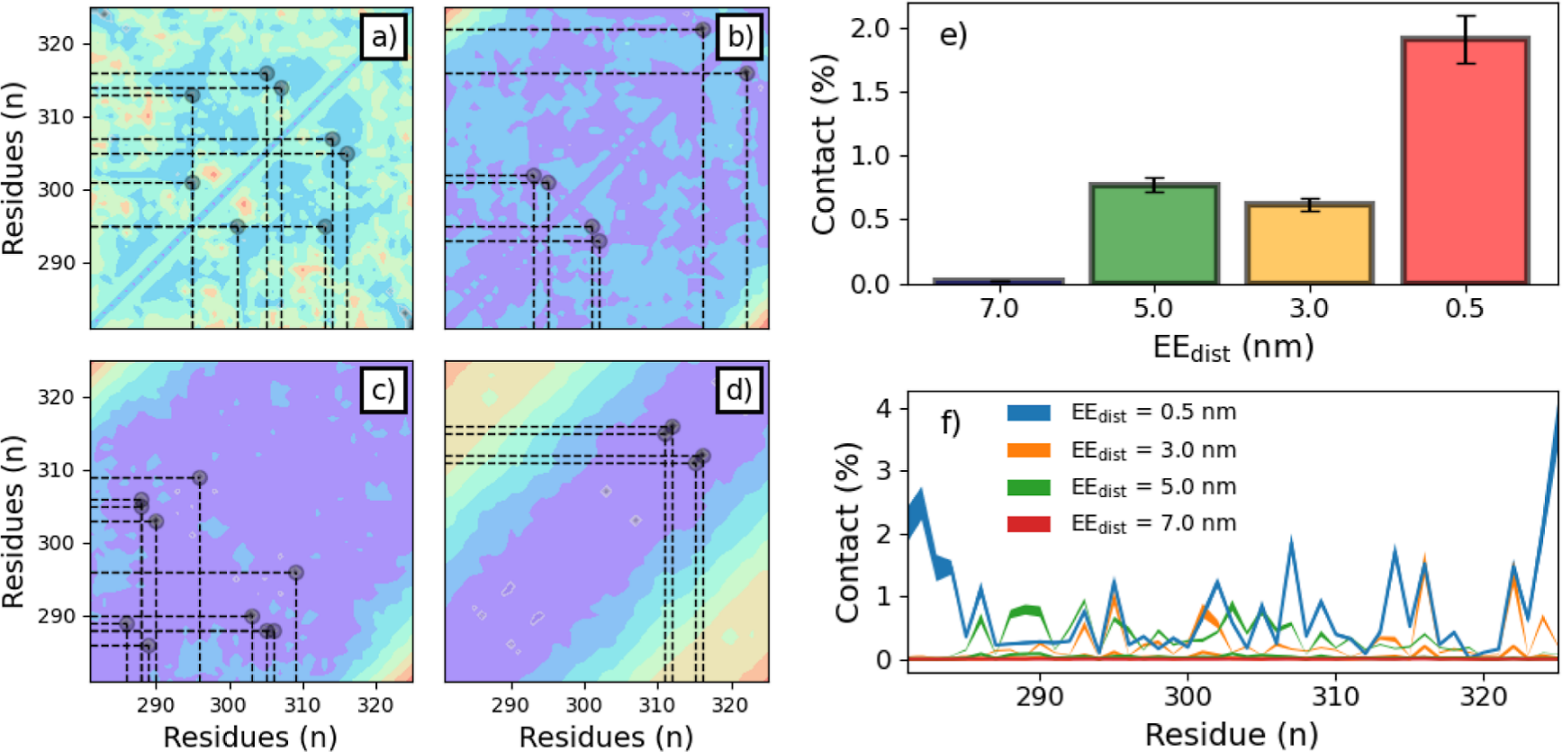
Minimum distances observed between α-carbons by residues in the restrained trajectories at 0.5 (a), 3.0 (b), 5.0 (c), and 7.0 nm (d) with dotted lines representing sites of significant contact. The average contacts by EE_dist_ (e) and by residue (f) are plotted for comparison.

**Figure 9:**
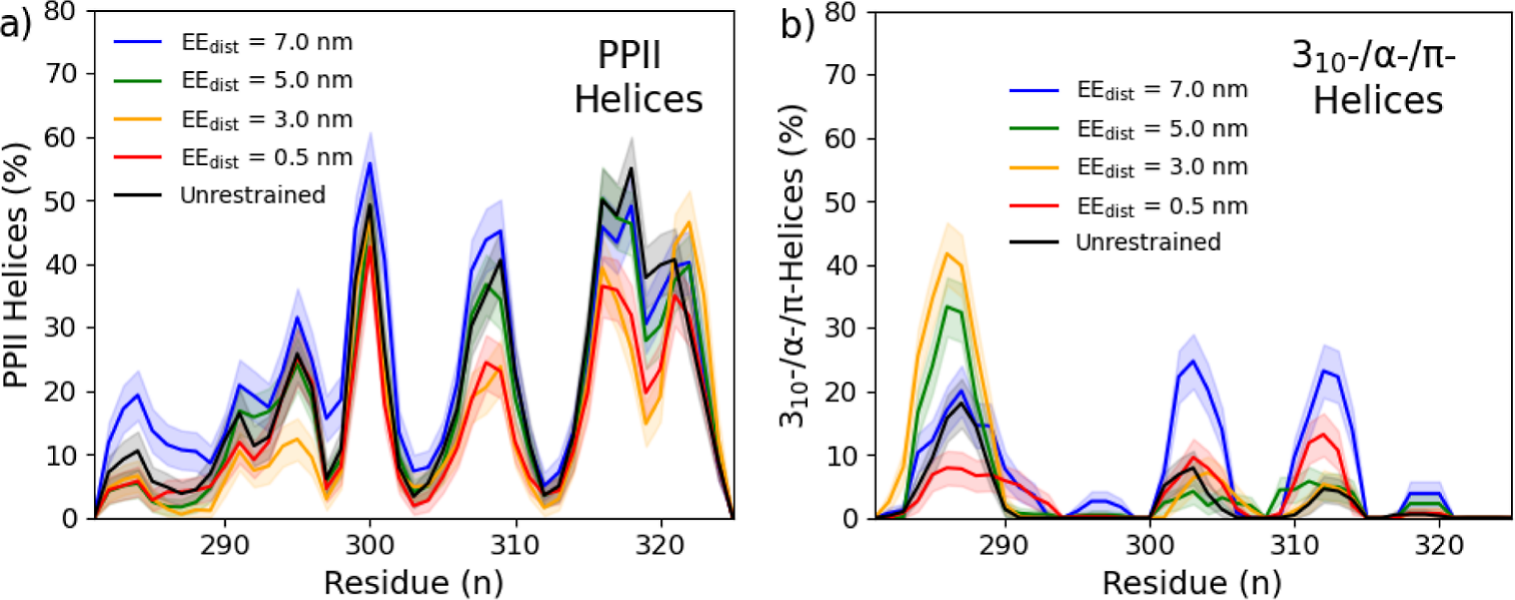
Relative distribution of PPII helices (a) and α-/3_10_-/π-helices (b) are displayed for each trajectory by residue for comparison.

Intramolecular interactions are crucial to understanding the influence of expansion on the disordered regions and how these regions operate differently between internal IDRs and IDPs. Figure 8a–d shows the contact probability for each restrained trajectory. The contacts between the end terminals are excluded from analysis, as they are in forcibly close proximity to each other. As expected, the number of interactions decreases as the trajectory is extended (Figure 8e). Comparing the contact map from the contracted PTL trajectory (Figure 8a) to the contact map from the unrestrained trajectory (Figure 5a), there is a region that indicates that intramolecular interactions are prohibited, whether the trajectory is restrained at any length or unrestrained, between Gly_298_ and Glu_302_. Interactions between these regions are discouraged by the structure, whether the simulation is restrained or freely moving. A possible explanation for this is binding in local adjacent residues, which “pinches” the sequence, prohibiting the residues from interacting. In the contracted trajectory, contacts are generally between distant residues, while more extended trajectories show favoritism for local residue interaction. This shift in the localization of the contacts may contribute to the transition in secondary structure from traditional α-helices and β-sheets to the more typically disordered associated PPII helices, which have been recorded to play a significant role in IDRs.^63^ The percent instances of PPII helices (Figure 9a) and more traditional 3_10_-/α-/π-helices (Figure 9b), as determined by DSSP-PPII, are plotted for analysis.

The presence of PP-II helices is observed most when the segment is extended and the least when contracted. Still, the change in the secondary structures is not found unilaterally across all residues. The helices forming between Glu_298_–Gly_302_ and Lys_320_–Leu_323_ are less impacted by the span of the region. In sharp contrast, the PP-II helices found between Lys_291_–Glu_298_, Arg_306_–Gln_310_, and Pro_316_–Lys_320_ seem diminished at smaller EE_dist_. For other helices, three regions present such structure; Arg_283_–Asn_288_, Gly_302_–Asn_305_, and Lys_310_–Ser_314_. Toward the N-terminus, this is most likely due to a partial secondary structure extending from the DBD, which, while not predicted in the IUPred (Figure 2), is observed in the EM structure and several rigid body models. The existence of these helices is favored at 3.0 and 5.0 nm and hindered at extreme EE_dist_. The other two regions seem transiently in the unrestrained and restrained simulations, with a significant preference (∼25%) when extended. These transient structures at which restraints are observed paint a picture of a dynamic region that is stabilized differently in different conformations.

**Figure 10:**
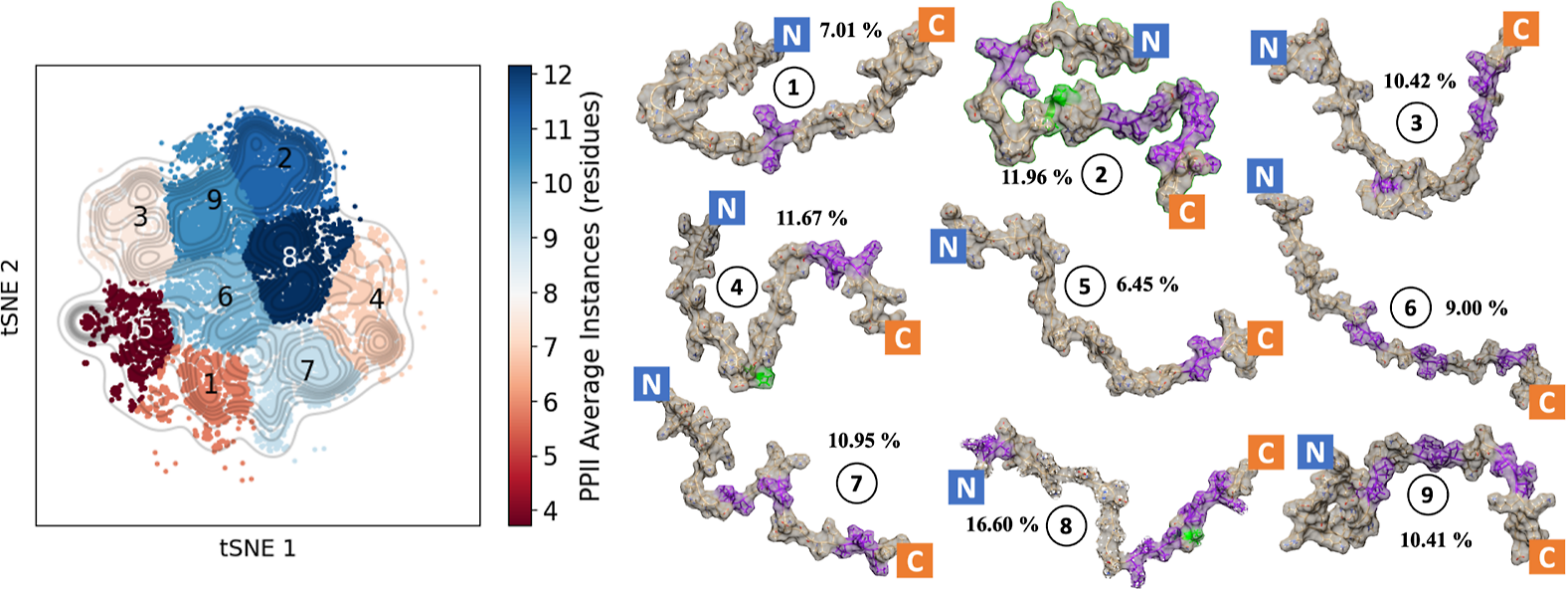
Graphical representation of the tSNE distributed landscape of the unrestrained trajectory along the ϕ and *psi* dihedral angles. Nine clusters were generated using the density-based OPTICS clustering algorithm, and the average number of residues with predicted PPII helices was calculated for color comparison on the plot (color bar). Graphical representation of the nine clusters can be seen (1–9) with PPII helices highlighted in purple and α-helices in green.

In terms of the structures formed, there is great difficulty in expressing the conformations available in an ensemble due to the tremendous number of degrees of freedom. Generating clusters requires a focus on preserving the relationship of data as it is expressed in lower dimensional space. Since PCA primarily preserves the variance in the data, a more complex DR technique is required for representative sampling, in this case, t-distributed Stochastic Neighbor Embedding (tSNE). For the unrestrained trajectory, tSNE DR was implemented using the ϕ and ψ dihedral angles as features, generating a reduced landscape (Figure 10). The different states in the landscape were clustered using density-based OPTICS clustering algorithms and plotted with colors according to the averaged propensity for PPII helices. Nine clusters were determined to describe the trajectory well (>95% of all total structures) with a silhouette score of ∼0.83. The comparison of the other cluster sizes, as well as the individual PPII propensities (Figure S10) based on a previous investigation into the importance of such structures for similar systems.^63^

The nine clusters from the unrestrained trajectory all contain some residues with PPII helices, as demonstrated by the DR and clustering (Figure 10) ranging from four to 12 residues on average. The distribution of the PPII helices varies, although there is a strong preference for the C-terminal and central residues. In three clusters, 2/4/8, some α-helices are also visible at the central residue. This structure has partially been observed in the electron microscopy^24^ of the monomeric form at this region.

### Experimental Assays and Models Assessment

**Figure 11:**
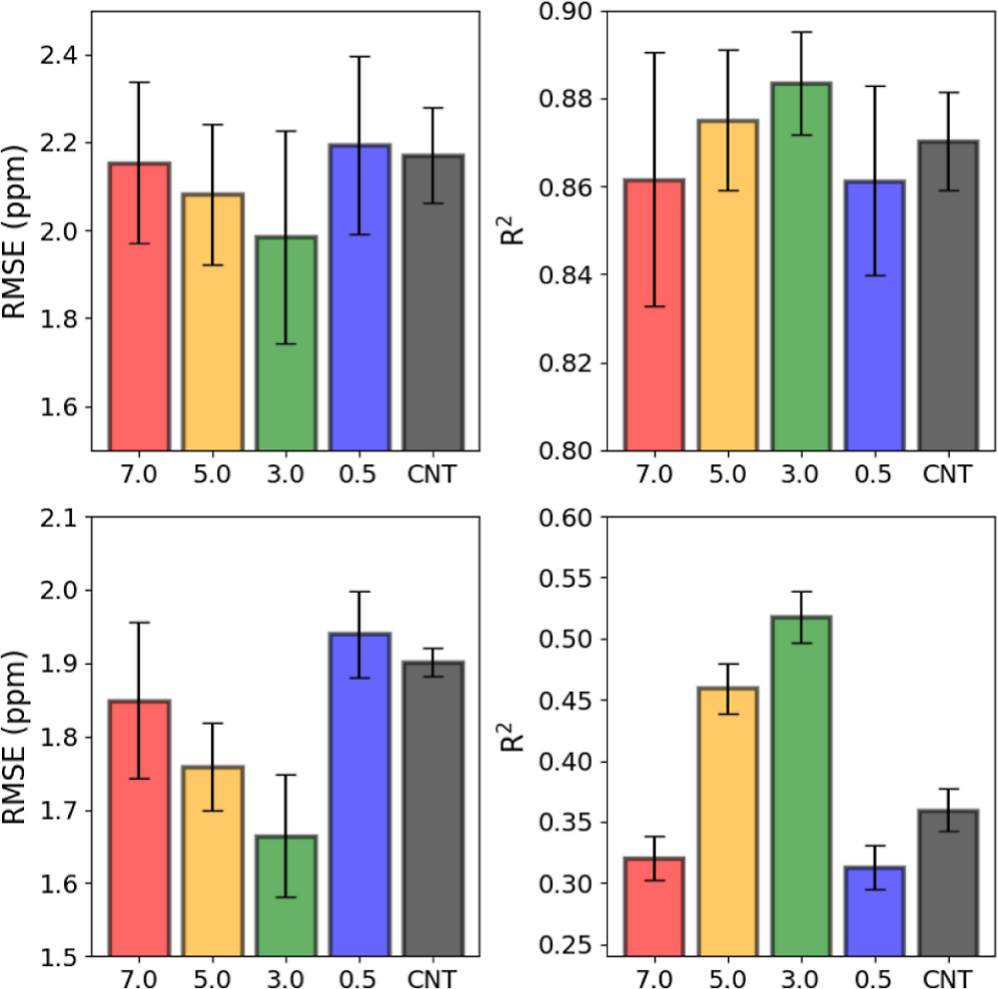
Comparison of predicted and experimentally obtained NMR CSs for the C_α_ and C′ atoms by root-mean-square error (RMSE) and correlation coefficient (*R*^2^) for residues in the PTL trajectories at various EE_dist_ and the unrestrained (CNT) trajectories. Error bars are generated, showing the standard deviation of the RMSE and RSQ for each frame compared to the experimental CSs.

The CS predictions from the various atoms in the residues were predicted using Sparta+ and compared (Figure 11) to the experimental NMR data^38^ for their correlation coefficient (*R*^2^) and RMSE. The experimental results errors are 0.015 ppm for ^1^H and 0.15 ppm for ^13^C and ^15^N. The C_β_ all produce such an excellent agreement (>0.99) that comparison between the models is ineffectual. The H_N_ CSs were not considered due to such a low correlation between experimental data and predicted CSs (<0.1), although the trend is the same as observed for other atoms (Figure 11). Additionally, the ^15^N CSs were also removed due to the nature by which Sparta+ derives its predictions. Since proline residues are particularly difficult to obtain experimentally, and Sparta+ is a neural network trained on experimental data, the program omits proline residues for ^15^N CSs.^55^ The PTL contains numerous proline residues; therefore, the ^15^N CSs were excluded.

The backbone carbons, however, strongly prefer the trajectory simulated with EE_dist_ = 3.0 nm, improving the RMSE by 0.22 nm from the unrestrained trajectory with C_α_ atoms and the *R*^2^ value from 0.33 to 0.56 for the C′ atoms. These results also agree with the EM structure observed (Figure 2) as the distance between the end terminals in the observed monomeric structure was 3.02 nm. The least agreement is found in the trajectories fully extended or fully contracted (0.5 and 7.0 nm), with the unrestrained trajectory somewhere in between. This tells us that sampling the trajectories with restrained terminals has a higher agreement regarding sampling local interactions and environments, drastically improving the model’s agreement with the experimental data.

**Figure 12:**
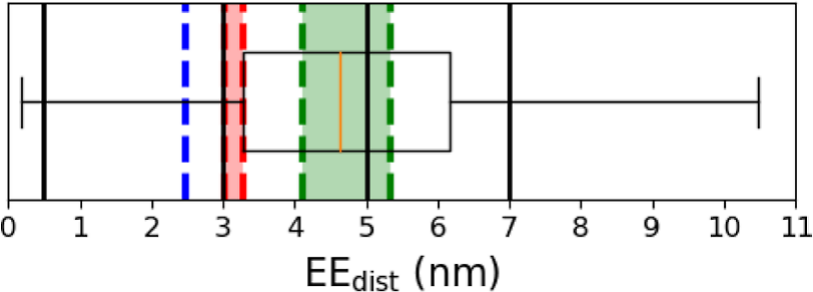
Distribution of PTL EE_dist_ from the unrestrained simulation depicted as a box plot with vertical lines representing the restrained trajectories. Dotted lines are drawn to represent the PTL’s span as observed from SAXS and EM for (red) p53 monomeric form unbound, (blue) p53 monomeric form bound to RNAPII (PDB: 6XRE), and (green) p53 tetrameric form bound to DNA (PDB: 7Y00).

Several rigid body models were generated/tested based on SAXS data collected of the protein in different bound states and compared to those generated from EM,^24,64,65^ as well as a predicted p53 monomeric form generated by AlphaFold.^66^ As seen in Table 2 the p53 protein can adopt multiple conformers depending on the bound state, and due to the limitations in the resolution of the experimental methods, these EE_dist_ values differ slightly. The observed general trend, however, is that p53 in its monomeric form expands upon tetramerization and DNA binding from ∼3 nm to ∼4.5 nm and contrasts upon binding to RNAPII to ∼2.4 nm. The AlphaFold prediction seems to underestimate the EE_dist_ by about 0.6 nm. The overall EE_dist_ distribution from the unrestrained trajectory can be seen as a box plot (Figure 12), with the restrained trajectories displayed as black lines and the experimental regions for the different bound states overlaid. Noticeably, the unrestrained trajectory explores conformations that are not observed in any experimentally derived methods (EE_dist_ > 6 nm), and this is reflected by the agreements seen between the NMR CSs (Figure 11).

**Table 2:** Distribution of PTL EE_dist_ Observed from Predicted Models and EM for Different States of the p53 Protein

method	state	binding	distance
AlphaFold	monomer	none	2.39 nm
EM	monomer	none	3.02 nm
SAXS	monomer	none	3.27 nm
EM	tetramer	DNA	4.10 nm
SAXS	tetramer	DNA	5.33 nm
EM	monomer	RNAP	2.47 nm

## Conclusions

The PTL region, when simulated as an IDP, is highly flexible and disordered, as shown by the predicted disorder (Figure 2) and by the Kratky plots from the simulation (Figure 3b). It is permitted to sample a wide variety of structures, predominantly PPII helices and random coils (Figure 4). Additionally, multiple likely sites of intramolecular interactions can be observed in the unrestrained trajectories (Figure 5), primarily consisting of proline residues. When the PTL was simulated with terminal restraints, the free energies were greatly shifted (Figure 6) with increased barriers and separation between the states observed at greater EE_dist_. The PTL also samples different secondary structures, with a preference for random coils and PPII helices at greater extended states (Figure 7). The intramolecular interactions also diverge, with a preference for distant and internal interactions in contracted states and more local, terminal interactions as the PTL is expanded (Figure 8). Overall these changes show a greatly altered conformational ensemble generated from the restrained trajectories to the unrestrained. Further investigation would be fruitful to test for the influence of the zwitterionic or neutral state of the termini, specifically for intermolecular interactions of the IDR. Testing these conformations against experimental CSs shows a strong preference in agreement for the conformations encapsulated by the restrained trajectory at EE_dist_ of 3.0 nm. This distance partially agrees with the structures obtained through SAXS rigid body modeling and EM (Figure 12).

## Supporting Information Available

RMSD plots from all the molecular dynamics trajectories, *R*_g_ plots and solvent-accessible surface area (SASA) plots for the restrained trajectories, shape factor analysis for the restrained trajectories, autocorrelation plots for all trajectories, free energy plots for the unrestrained trajectory separated by individual replicates, EE_dist_ distances and *R*_g_ plots for the different replicates of the unrestrained trajectory, tSNE dimensionally reduced landscapes for the restrained trajectories, CS prediction errors for all trajectories by residue, root-mean-square fluctuation (RMSF) plots for each trajectory, and the hydrogen bonding observed by trajectory per residue (PDF)

## References

[ref1] Rutkowski R.; Hofmann K.; Gartner A. Phylogeny and function of the invertebrate p53 superfamily. Cold Spring Harbor Perspect. Biol. 2010, 2, a001131 10.1101/cshperspect.a001131. PMC2890203 20595397

[ref2] Vousden K. H. Activation of the p53 tumor suppressor protein. Biochim. Biophys. Acta, Rev. Cancer 2002, 1602, 47–59. 10.1016/S0304-419X(02)00035-5. 11960694

[ref3] Ryan K. M.; Phillips A. C.; Vousden K. H. Regulation and function of the p53 tumor suppressor protein. Curr. Opin. Cell Biol. 2001, 13, 332–337. 10.1016/S0955-0674(00)00216-7. 11343904

[ref4] Oren M. Regulation of the p53 tumor suppressor protein. J. Biol. Chem. 1999, 274, 36031–36034. 10.1074/jbc.274.51.36031. 10593882

[ref5] Surget S.; Khoury M. P.; Bourdon J.-C. Uncovering the role of p53 splice variants in human malignancy: a clinical perspective. OncoTargets Ther. 2013, 7, 57–68. 10.2147/OTT.S53876. PMC3872270 24379683

[ref6] Kato S.; Han S.-Y.; Liu W.; Otsuka K.; Shibata H.; Kanamaru R.; Ishioka C. Understanding the function–structure and function–mutation relationships of p53 tumor suppressor protein by high-resolution missense mutation analysis. Proc. Natl. Acad. Sci. U.S.A. 2003, 100, 8424–8429. 10.1073/pnas.1431692100. 12826609 PMC166245

[ref7] Ozaki T.; Nakagawara A. Role of p53 in cell death and human cancers. Cancers 2011, 3, 994–1013. 10.3390/cancers3010994. 24212651 PMC3756401

[ref8] Rizzotto D.; Englmaier L.; Villunger A. At a crossroads to cancer: how p53-induced cell fate decisions secure genome integrity. Int. J. Mol. Sci. 2021, 22, 10883 10.3390/ijms221910883. 34639222 PMC8509445

[ref9] Belyi V. A.; Ak P.; Markert E.; Wang H.; Hu W.; Puzio-Kuter A.; Levine A. J. The origins and evolution of the p53 family of genes. Cold Spring Harbor Perspect. Biol. 2009, 2, a001198 10.1101/cshperspect.a001198. PMC2869528 20516129

[ref10] Belyi V. A.; Ak P.; Markert E.; Wang H.; Hu W.; Puzio-Kuter A.; Levine A. J. The origins and evolution of the p53 family of genes. Cold Spring Harbor Perspect. Biol. 2010, 2, a001198 10.1101/cshperspect.a001198. PMC2869528 20516129

[ref11] Steele R. J.; Thompson A. M.; Hall P. A.; Lane D. P. The p53 tumour suppressor gene. Br. J. Surg. 2003, 85, 1460–1467. 10.1046/j.1365-2168.1998.00910.x. 9823903

[ref12] Petitjean A.; Achatz M. I.; Borresen-Dale A. L.; Hainaut P.; Olivier M. TP53 mutations in human cancers: Functional selection and impact on cancer prognosis and outcomes. Oncogene 2007, 26, 2157–2165. 10.1038/sj.onc.1210302. 17401424

[ref13] Marei H. E.; Althani A.; Afifi N.; Hasan A.; Caceci T.; Pozzoli G.; Morrione A.; Giordano A.; Cenciarelli C. p53 signaling in cancer progression and therapy. Cancer Cell Int. 2021, 21, 703 10.1186/s12935-021-02396-8. 34952583 PMC8709944

[ref14] Natan E.; Baloglu C.; Pagel K.; Freund S. M.; Morgner N.; Robinson C. V.; Fersht A. R.; Joerger A. C. Interaction of the p53 DNA-binding domain with its N-terminal extension modulates the stability of the P53 tetramer. J. Mol. Biol. 2011, 409, 358–368. 10.1016/j.jmb.2011.03.047. 21457718 PMC3176915

[ref15] Han C. W.; Lee H. N.; Jeong M. S.; Park S. Y.; Jang S. B. Structural basis of the p53 DNA binding domain and Puma Complex. Biochem. Biophys. Res. Commun. 2021, 548, 39–46. 10.1016/j.bbrc.2021.02.049. 33631672

[ref16] Marques M. A.; de Oliveira G. A.; Silva J. L. The chameleonic behavior of p53 in health and disease: the transition from a client to an aberrant condensate scaffold in cancer. Essays Biochem. 2022, 66, 1023–1033. 10.1042/EBC20220064. 36350030

[ref17] Baughman H. E.; Narang D.; Chen W.; Villagrán Suárez A. C.; Lee J.; Bachochin M. J.; Gunther T. R.; Wolynes P. G.; Komives E. A. An intrinsically disordered transcription activation domain increases the DNA binding affinity and reduces the specificity of NFκB p50/RelA. J. Biol. Chem. 2022, 298, 102349 10.1016/j.jbc.2022.102349. 35934050 PMC9440430

[ref18] Baptiste N.; Friedlander P.; Chen X.; Prives C. The proline-rich domain of p53 is required for cooperation with anti-neoplastic agents to promote apoptosis of tumor cells. Oncogene 2002, 21, 9–21. 10.1038/sj.onc.1205015. 11791172

[ref19] Jenkins L. M.; Durell S. R.; Mazur S. J.; Appella E. P53 N-terminal phosphorylation: A defining layer of complex regulation. Carcinogenesis 2012, 33, 1441–1449. 10.1093/carcin/bgs145. 22505655 PMC3499055

[ref20] Luciani M.; Hutchins J. R.; Zheleva D.; Hupp T. R. The C-terminal regulatory domain of P53 contains a functional docking site for cyclin A. J. Mol. Biol. 2000, 300, 503–518. 10.1006/jmbi.2000.3830. 10884347

[ref21] Chène P. The role of tetramerization in p53 function. Oncogene 2001, 20, 2611–2617. 10.1038/sj.onc.1204373. 11420672

[ref22] Jeffrey P. D.; Gorina S.; Pavletich N. P. Crystal structure of the tetramerization domain of the p53 tumor suppressor at 1.7 angstroms. Science 1995, 267, 1498–1502. 10.1126/science.7878469. 7878469

[ref23] Gencel-Augusto J.; Lozano G. P53 tetramerization: At the center of the dominant-negative effect of mutant p53. Genes Dev. 2020, 34, 1128–1146. 10.1101/gad.340976.120. 32873579 PMC7462067

[ref24] Solares M. J.; Jonaid G. M.; Luqiu W. Y.; Berry S.; Khadela J.; Liang Y.; Evans M. C.; Pridham K. J.; Dearnaley W. J.; Sheng Z.; Kelly D. F. High-Resolution Imaging of Human Cancer Proteins Using Microprocessor Materials. ChemBioChem 2022, 23, e202200310 10.1002/cbic.202200310. 35789183 PMC9574649

[ref25] Dosztanyi Z.; Csizmok V.; Tompa P.; Simon I. IUPred: web server for the prediction of intrinsically unstructured regions of proteins based on estimated energy content. Bioinformatics 2005, 21, 3433–3434. 10.1093/bioinformatics/bti541. 15955779

[ref26] Gopal S. M.; Wingbermühle S.; Schnatwinkel J.; Juber S.; Herrmann C.; Schäfer L. V. Conformational preferences of an intrinsically disordered protein domain: A case study for modern force fields. J. Phys. Chem. B 2020, 125, 24–35. 10.1021/acs.jpcb.0c08702. 33382616

[ref27] Na J.-H.; Lee W.-K.; Yu Y. G. How do we study the dynamic structure of unstructured proteins: a case study on Nopp140 as an example of a large, intrinsically disordered protein. Int. J. Mol. Sci. 2018, 19, 381 10.3390/ijms19020381. 29382046 PMC5855603

[ref28] Stanley N.; Esteban-Martín S.; De Fabritiis G. Progress in studying intrinsically disordered proteins with atomistic simulations. Prog. Biophys. Mol. Biol. 2015, 119, 47–52. 10.1016/j.pbiomolbio.2015.03.003. 25814479

[ref29] Akbayrak I. Y.; Caglayan S. I.; Ozcan Z.; Uversky V. N.; Coskuner-Weber O. Current challenges and limitations in the studies of intrinsically disordered proteins in neurodegenerative diseases by computer simulations. Curr. Alzheimer Res. 2021, 17, 805–818. 10.2174/1567205017666201109094908. 33167839

[ref30] Carballo-Pacheco M.; Strodel B. Comparison of force fields for Alzheimer’s A: A case study for intrinsically disordered proteins. Protein Sci. 2017, 26, 174–185. 10.1002/pro.3064. 27727496 PMC5275744

[ref31] Mu J.; Liu H.; Zhang J.; Luo R.; Chen H.-F. Recent force field strategies for intrinsically disordered proteins. J. Chem. Inf. Model. 2021, 61, 1037–1047. 10.1021/acs.jcim.0c01175. 33591749 PMC8256680

[ref32] Babu M. M.; van der Lee R.; de Groot N. S.; Gsponer J. Intrinsically disordered proteins: regulation and disease. Curr. Opin. Struct. Biol. 2011, 21, 432–440. 10.1016/j.sbi.2011.03.011. 21514144

[ref33] van der Lee R.; Buljan M.; Lang B.; Weatheritt R. J.; Daughdrill G. W.; Dunker A. K.; Fuxreiter M.; Gough J.; Gsponer J.; Jones D. T.; Kim P. M.; Kriwacki R. W.; Oldfield C. J.; Pappu R. V.; et al. Classification of intrinsically disordered regions and proteins. Chem. Rev. 2014, 114, 6589–6631. 10.1021/cr400525m. 24773235 PMC4095912

[ref34] Janin J.; Sternberg M. J. Protein flexibility, not disorder, is intrinsic to molecular recognition. F1000Prime Rep. 2013, 5, 2 10.3410/b5-2. PMC3542771 23361309

[ref35] Marsh J. A.; Teichmann S. A.; Forman-Kay J. D. Probing the diverse landscape of protein flexibility and binding. Curr. Opin. Struct. Biol. 2012, 22, 643–650. 10.1016/j.sbi.2012.08.008. 22999889

[ref36] Williams R.; Obradovic Z.; Mathura V.; Braun W.; Garner E.; Young J.; Takayama S.; Brown C. J.; Dunker A. K. Biocomputing 2001; World Scientific, 2000, pp 89–100. 10.1142/9789814447362_0010 11262981

[ref37] Kim D.-H.; Han K.-H. Transient secondary structures as general target-binding motifs in intrinsically disordered proteins. Int. J. Mol. Sci. 2018, 19, 3614 10.3390/ijms19113614. 30445805 PMC6275026

[ref38] Mandal R.; Kohoutova K.; Petrvalska O.; Horvath M.; Srb P.; Veverka V.; Obsilova V.; Obsil T. FOXO4 interacts with p53 TAD and CRD and inhibits its binding to DNA. Protein Sci. 2022, 31, e4287 10.1002/pro.4287. 35481640 PMC8994487

[ref39] Abraham M. J.; Murtola T.; Schulz R.; Páll S.; Smith J. C.; Hess B.; Lindahl E. GROMACS: High performance molecular simulations through multi-level parallelism from laptops to supercomputers. SoftwareX 2015, 1–2, 19–25. 10.1016/j.softx.2015.06.001.

[ref40] Pronk S.; Páll S.; Schulz R.; Larsson P.; Bjelkmar P.; Apostolov R.; Shirts M. R.; Smith J. C.; Kasson P. M.; Van Der Spoel D.; Hess B.; Lindahl E. GROMACS 4.5: a high-throughput and highly parallel open source molecular simulation toolkit. Bioinformatics 2013, 29, 845–854. 10.1093/bioinformatics/btt055. 23407358 PMC3605599

[ref41] Hess B.; Kutzner C.; Van Der Spoel D.; Lindahl E. GROMACS 4: algorithms for highly efficient, load-balanced, and scalable molecular simulation. J. Chem. Theory Comput. 2008, 4, 435–447. 10.1021/ct700301q. 26620784

[ref42] Berendsen H. J.; van der Spoel D.; van Drunen R. GROMACS: A message-passing parallel molecular dynamics implementation. Comput. Phys. Commun. 1995, 91, 43–56. 10.1016/0010-4655(95)00042-e.

[ref43] Lindorff-Larsen K.; Piana S.; Palmo K.; Maragakis P.; Klepeis J. L.; Dror R. O.; Shaw D. E. Improved side-chain torsion potentials for the Amber ff99SB protein force field. Proteins: Struct., Funct., Bioinf. 2010, 78, 1950–1958. 10.1002/prot.22711. PMC2970904 20408171

[ref44] Yang S.; Liu H.; Zhang Y.; Lu H.; Chen H. Residue-specific force field improving the sample of intrinsically disordered proteins and folded proteins. J. Chem. Inf. Model. 2019, 59, 4793–4805. 10.1021/acs.jcim.9b00647. 31613621

[ref45] Henriques J.; Cragnell C.; Skepo M. Molecular dynamics simulations of intrinsically disordered proteins: force field evaluation and comparison with experiment. J. Chem. Theory Comput. 2015, 11, 3420–3431. 10.1021/ct501178z. 26575776

[ref46] Henriques J.; Skepö M. Molecular dynamics simulations of intrinsically disordered proteins: on the accuracy of the TIP4P-D water model and the representativeness of protein disorder models. J. Chem. Theory Comput. 2016, 12, 3407–3415. 10.1021/acs.jctc.6b00429. 27243806

[ref47] Rieloff E.; Skepö M. Molecular dynamics simulations of phosphorylated intrinsically disordered proteins: A force field comparison. Int. J. Mol. Sci. 2021, 22, 10174 10.3390/ijms221810174. 34576338 PMC8470740

[ref48] Chen W.; Shi C.; MacKerell A. D.; Shen J. Conformational dynamics of two natively unfolded fragment peptides: comparison of the AMBER and CHARMM force fields. J. Phys. Chem. B 2015, 119, 7902–7910. 10.1021/acs.jpcb.5b02290. 26020564 PMC4685472

[ref49] Zapletal V.; Mládek A.; Melková K.; Louša P.; Nomilner E.; Jaseňáková Z.; Kubáň V.; Makovická M.; Laníková A.; Žídek L.; Hritz J. Choice of force field for proteins containing structured and intrinsically disordered regions. Biophys. J. 2020, 118, 1621–1633. 10.1016/j.bpj.2020.02.019. 32367806 PMC7136338

[ref50] Hanwell M. D.; Curtis D. E.; Lonie D. C.; Vandermeersch T.; Zurek E.; Hutchison G. R. Avogadro: an advanced semantic chemical editor, visualization, and analysis platform. J. Cheminf. 2012, 4, 17 10.1186/1758-2946-4-17. PMC3542060 22889332

[ref51] Ozenne V.; Bauer F.; Salmon L.; Huang J.-r.; Jensen M. R.; Segard S.; Bernadó P.; Charavay C.; Blackledge M. Flexible-Meccano: a tool for the generation of explicit ensemble descriptions of intrinsically disordered proteins and their associated experimental observables. Bioinformatics 2012, 28, 1463–1470. 10.1093/bioinformatics/bts172. 22613562

[ref52] McGibbon R. T.; Beauchamp K. A.; Harrigan M. P.; Klein C.; Swails J. M.; Hernández C. X.; Schwantes C. R.; Wang L.-P.; Lane T. J.; Pande V. S. MDTraj: a modern open library for the analysis of molecular dynamics trajectories. Biophys. J. 2015, 109, 1528–1532. 10.1016/j.bpj.2015.08.015. 26488642 PMC4623899

[ref53] Svergun D.; Barberato C.; Koch M. H. CRYSOL–a program to evaluate X-ray solution scattering of biological macromolecules from atomic coordinates. J. Appl. Crystallogr. 1995, 28, 768–773. 10.1107/S0021889895007047.

[ref54] Chebrek R.; Leonard S.; de Brevern A. G.; Gelly J.-C. PolyprOnline: polyproline helix II and secondary structure assignment database. Database 2014, 2014, bau102 10.1093/database/bau102. 25380779 PMC4224144

[ref55] Shen Y.; Bax A. SPARTA+: a modest improvement in empirical NMR chemical shift prediction by means of an artificial neural network. J. Biomol. NMR 2010, 48, 13–22. 10.1007/s10858-010-9433-9. 20628786 PMC2935510

[ref56] Scherer M. K.; Trendelkamp-Schroer B.; Paul F.; Pérez-Hernández G.; Hoffmann M.; Plattner N.; Wehmeyer C.; Prinz J.-H.; Noé F. PyEMMA 2: A software package for estimation, validation, and analysis of Markov models. J. Chem. Theory Comput. 2015, 11, 5525–5542. 10.1021/acs.jctc.5b00743. 26574340

[ref57] Campos S. R.; Baptista A. M. Conformational analysis in a multidimensional energy landscape: study of an arginylglutamate repeat. J. Phys. Chem. B 2009, 113, 15989–16001. 10.1021/jp902991u. 19778072

[ref58] Kramer O. Scikit-learn. In Machine learning for evolution strategies; Springer 2016; Vol. 20; pp 45–53. 10.1007/978-3-319-33383-0_5.

[ref59] Konarev P. V.; Volkov V. V.; Sokolova A. V.; Koch M. H.; Svergun D. I. PRIMUS: a Windows PC-based system for small-angle scattering data analysis. J. Appl. Crystallogr. 2003, 36, 1277–1282. 10.1107/S0021889803012779.

[ref60] DeLano W. L. Pymol: An open-source molecular graphics tool. CCP4 Newsl. Protein Crystallogr. 2002, 40, 82–92.

[ref61] Pettersen E. F.; Goddard T. D.; Huang C. C.; Couch G. S.; Greenblatt D. M.; Meng E. C.; Ferrin T. E. UCSF Chimera—a visualization system for exploratory research and analysis. J. Comput. Chem. 2004, 25, 1605–1612. 10.1002/jcc.20084. 15264254

[ref62] Koder Hamid M.; Månsson L. K.; Meklesh V.; Persson P.; Skepö M. Molecular dynamics simulations of the adsorption of an intrinsically disordered protein: Force field and water model evaluation in comparison with experiments. Front. Mol. Biosci. 2022, 9, 958175 10.3389/fmolb.2022.958175. 36387274 PMC9644065

[ref63] Jephthah S.; Pesce F.; Lindorff-Larsen K.; Skepo M. Force field effects in simulations of flexible peptides with varying polyproline II propensity. J. Chem. Theory Comput. 2021, 17, 6634–6646. 10.1021/acs.jctc.1c00408. 34524800 PMC8515809

[ref64] Melero R.; Rajagopalan S.; Lázaro M.; Joerger A. C.; Brandt T.; Veprintsev D. B.; Lasso G.; Gil D.; Scheres S. H.; Carazo J. M.; Fersht A. R.; Valle M. Electron microscopy studies on the quaternary structure of p53 reveal different binding modes for p53 tetramers in complex with DNA. Proc. Natl. Acad. Sci. U.S.A. 2011, 108, 557–562. 10.1073/pnas.1015520107. 21178074 PMC3021029

[ref65] Okorokov A. L.; Orlova E. V. Structural biology of the p53 tumor suppressor. Curr. Opin. Struct. Biol. 2009, 19, 197–202. 10.1016/j.sbi.2009.02.003. 19286366

[ref66] Skolnick J.; Gao M.; Zhou H.; Singh S. AlphaFold 2: why it works and its implications for understanding the relationships of protein sequence, structure, and function. J. Chem. Inf. Model. 2021, 61, 4827–4831. 10.1021/acs.jcim.1c01114. 34586808 PMC8592092

