# Supplementary material for: Exploring the Role of Globular Domain Locations on an Intrinsically Disordered Region of p53: A Molecular Dynamics Investigation
Source: J Chem Theory Comput. 2024 Jan 17;20(3):1423–33. doi: 10.1021/acs.jctc.3c00971 (PMC10867847; doi:10.1021/acs.jctc.3c00971)
Supplement: Supplementary file 1 — ct3c00971_si_001.pdf [file ct3c00971_si_001.pdf]

Supporting Information for Exploring the Role of Globular Domain Locations on an Intrinsically Disordered Region of p53: A Molecular Dynamics Investigation

Michael J. Bakker,† Henrik V. Sørensen,‡ and Marie Skepö*,‡,¶

†*Faculty of Pharmacy in Hradec Králové, Charles University, Akademika Heyrovského 1203/8, 500 05 Hradec Králové, Czech Republic*

‡*Division of Computational Chemistry, Department of Chemistry, Lund University, P.O. Box 124, SE-221 00, Lund, Sweden*

¶*LINXS - Institute of Advanced Neutron and X-ray Science, Scheelevägen 19, SE-233 70, Lund, Sweden*

E-mail: marie.skepo@compchem.lu.se

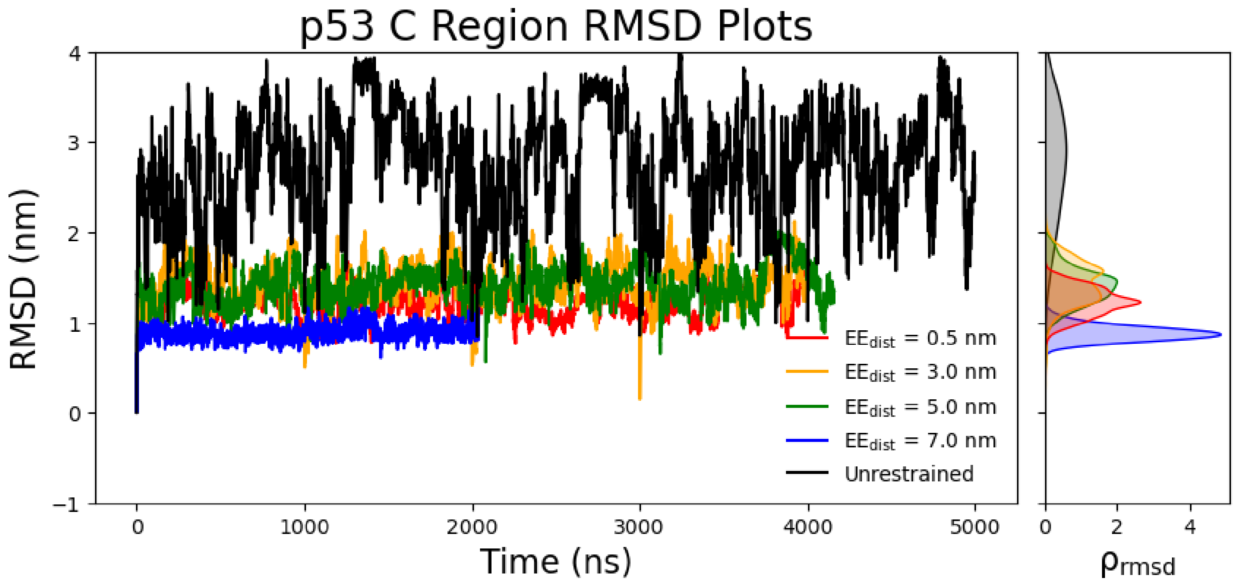

Figure S1: Root-mean-square distance (RMSD) plots and kernel density estimate (KDE) of the various trajectories showing convergence and variation in conformations for the unrestrained and restrained trajectories at different end-to-end distances (EE_{dist}).

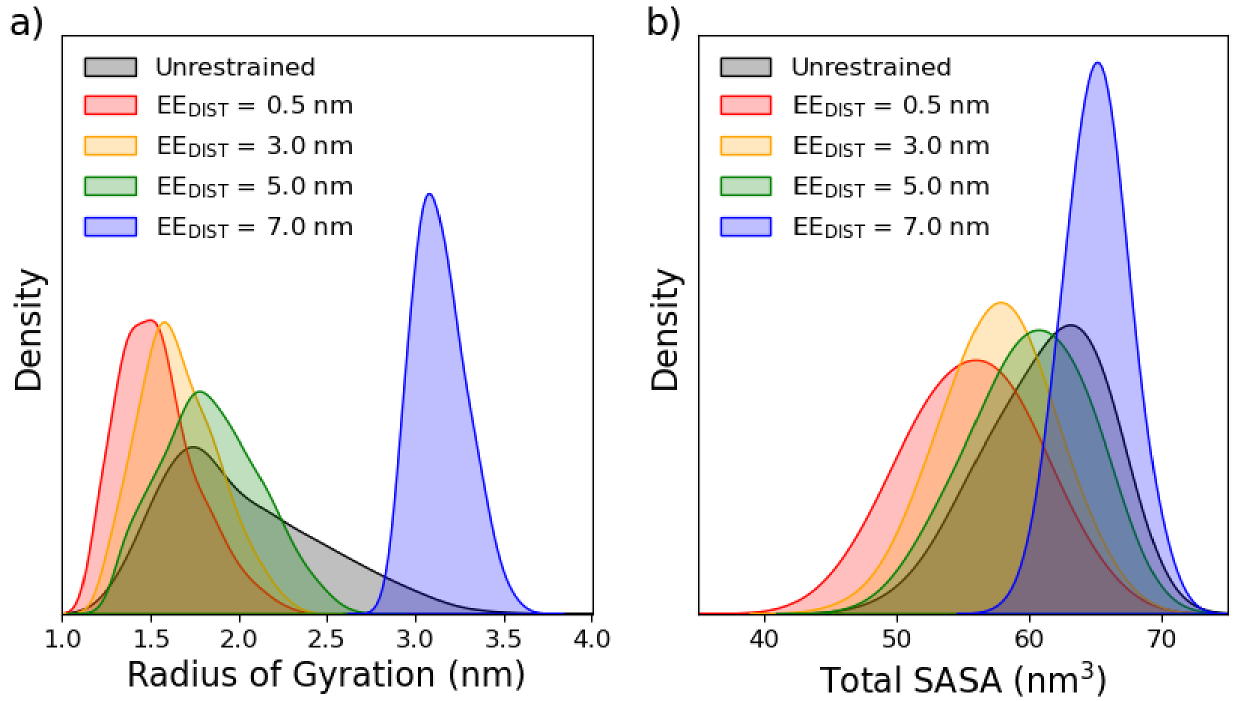

Figure S2: Kernel density estimate (KDE) plots of the radius of gyration (a) and the solvent-accessible surface area (SASA) (b) from the pre-tetramerizational loop trajectories unrestrained and restrained at different end-to-end distances (EE_{dist}).

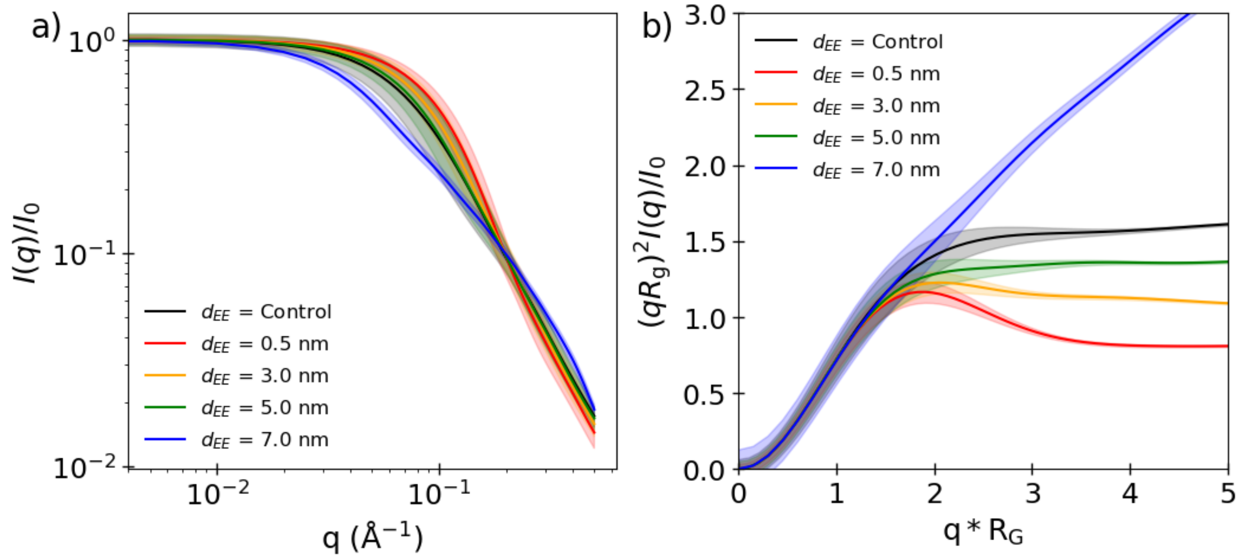

Figure S3: SAXS intensity curve $I(q)/I_0$ vs q (a) and the dimensionless Kratky plot (b) for the pre-tetramerizational loop trajectories unrestrained and restrained at different EE_{dist}.

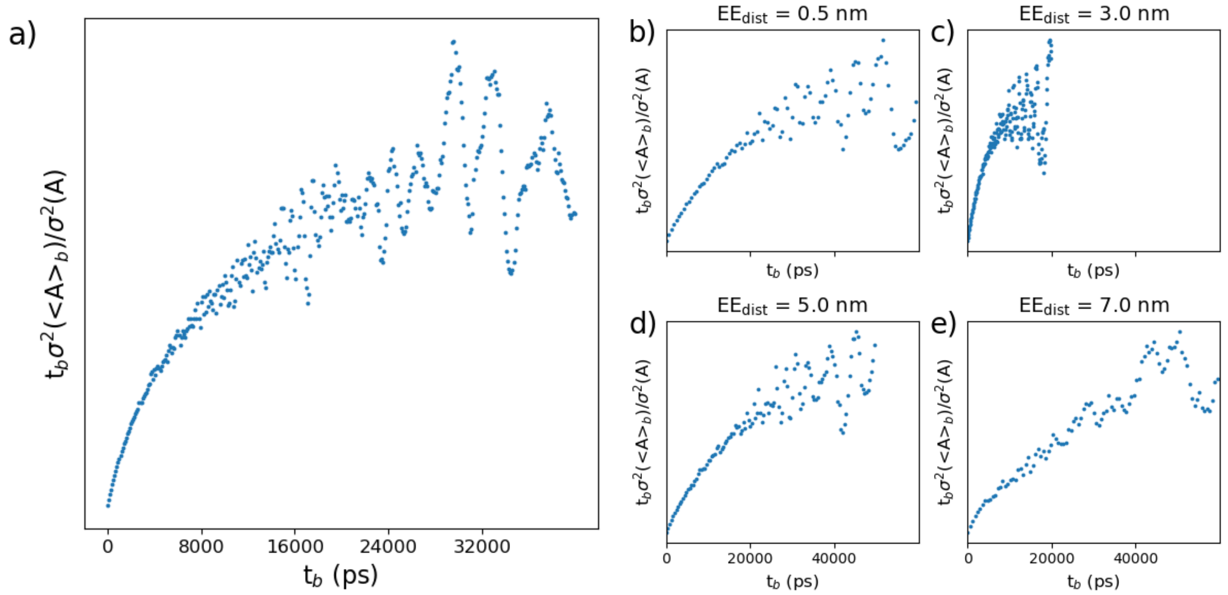

Figure S4: Autocorrelation assessment comparing the block sizes (t_b) to the statistical inefficiency for the unrestrained (a) trajectory, and the restrained trajectories (b-e) at different end-to-end distances (EE_{dist}).

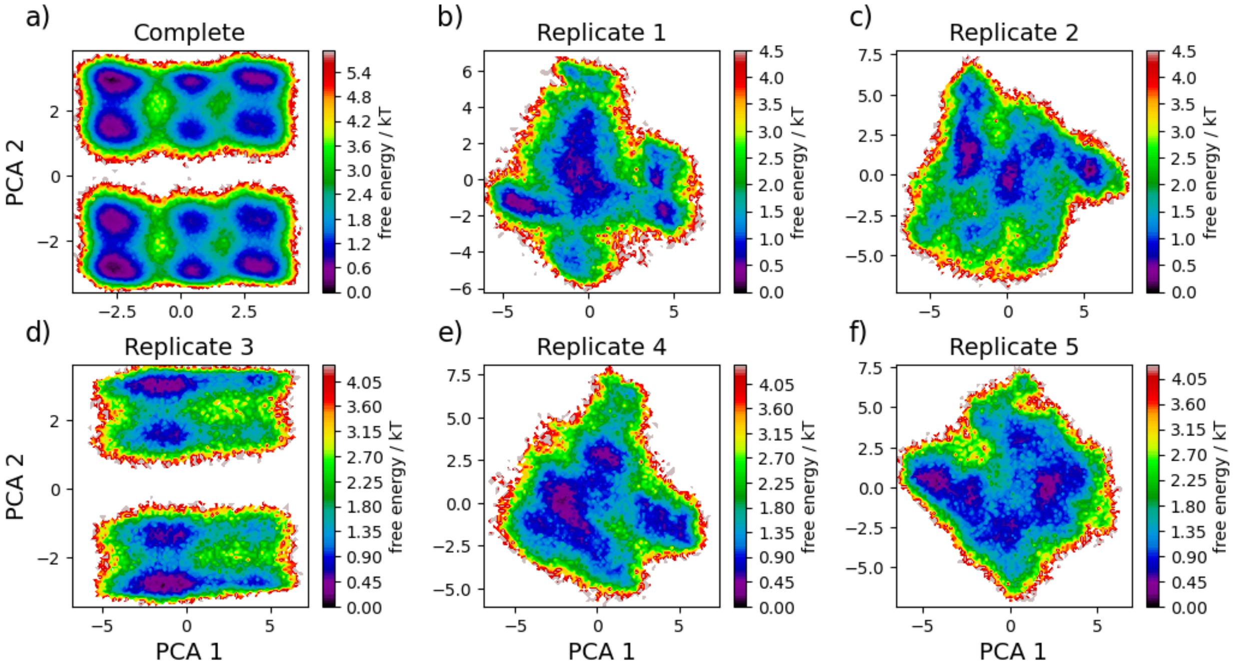

Figure S5: Free energy plots from a dimensionality reduction (PCA) for the complete pre-tetramerizational loop unrestrained trajectory and the 5 microsecond replicates for comparison and convergence.

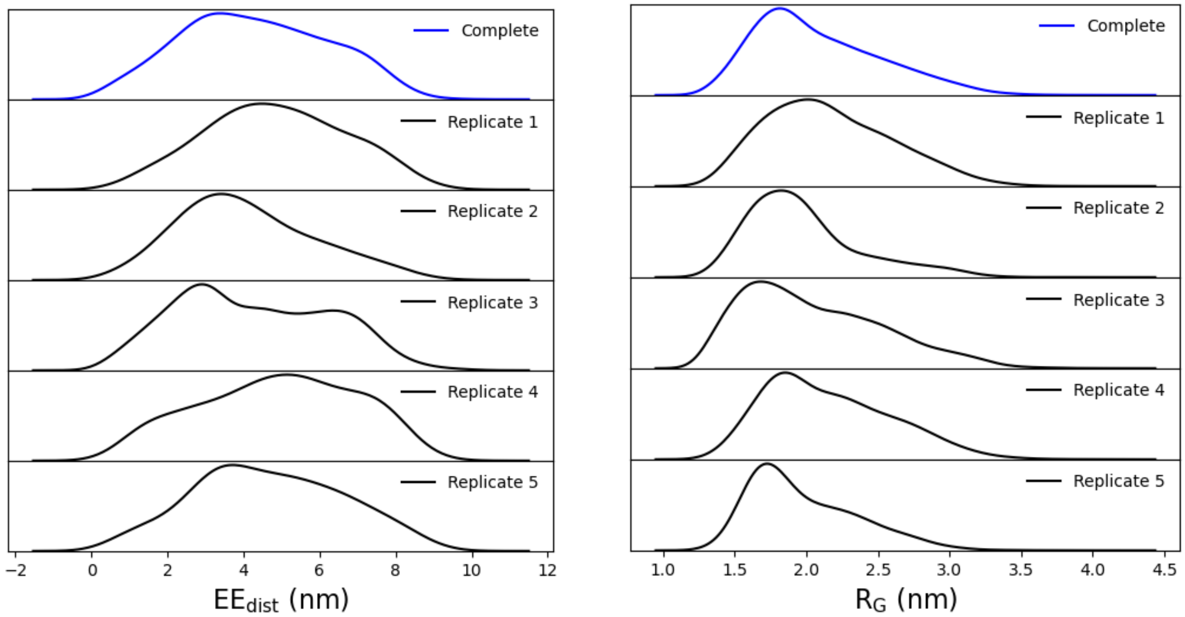

Figure S6: Replicate analysis for the unrestrained pre-tetramerizational loop trajectories interpreted for their end-to-end distance (EE_{dist}) and radius of gyration (R_G), respectively.

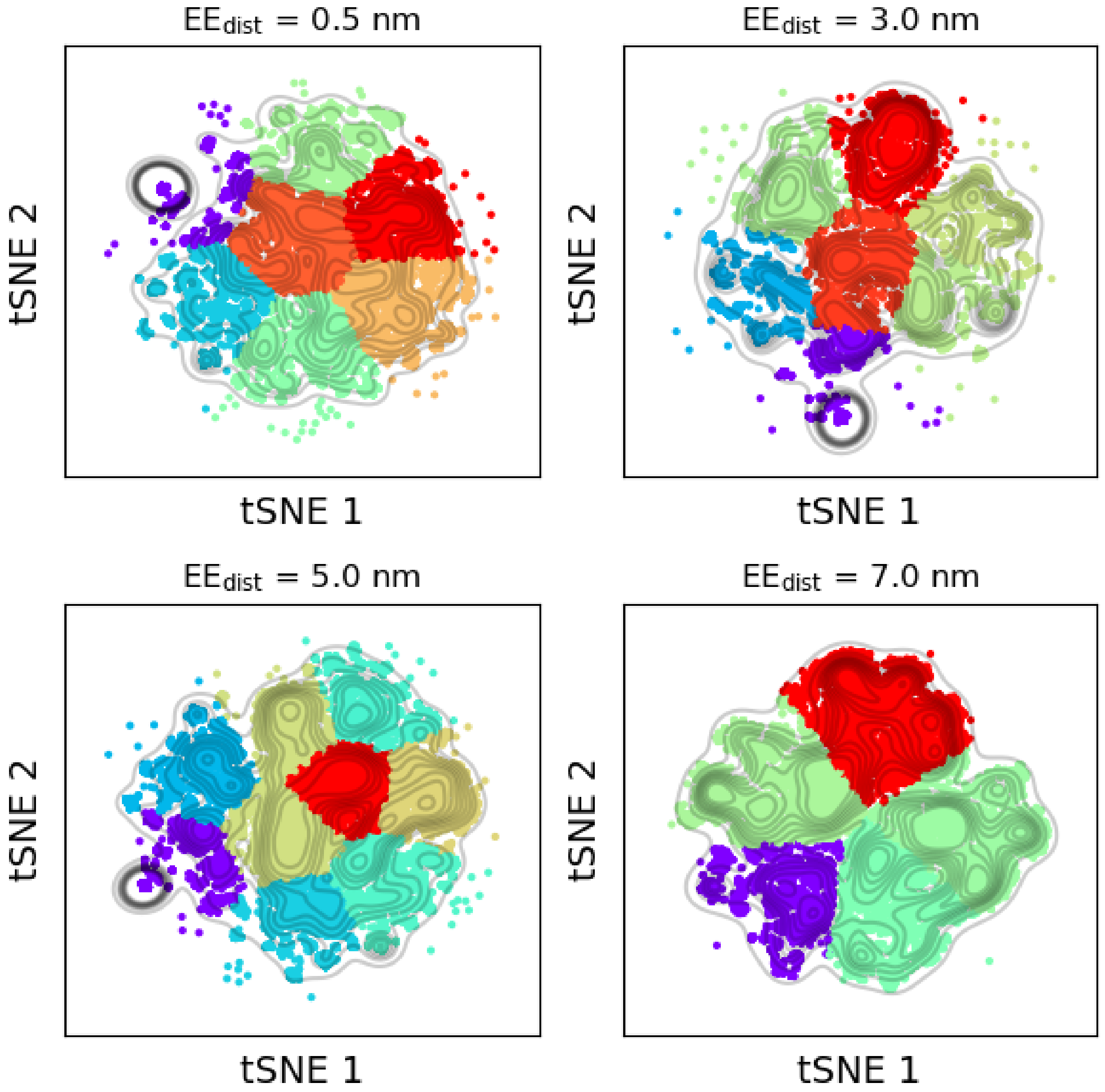

Figure S7: tSNE dimensionality reduction and *DBSCAN* clustering of the dihedral angles in the pre-tetramerizational loop for the restrained trajectories at different end-to-end distances (EE_{dist}), respectively.

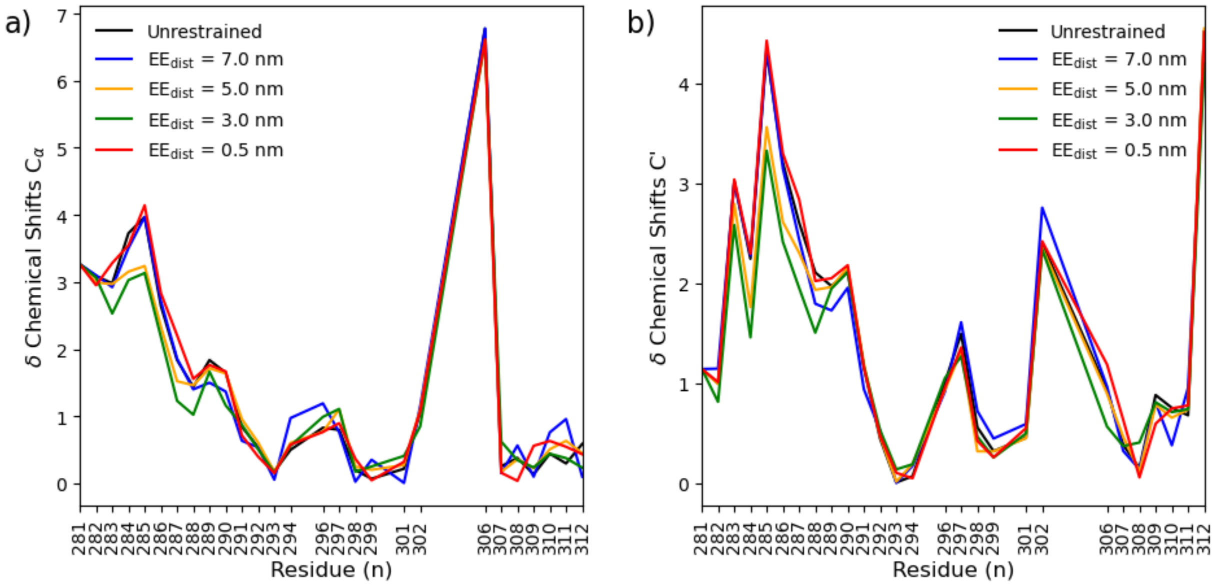

Figure S8: Averaged chemical shift predictions from Sparta+ difference from experimental value by residue for the C_α (a) and C' (b) ^{13}C NMR at different end-to-end distances (EE_{dist}), respectively.

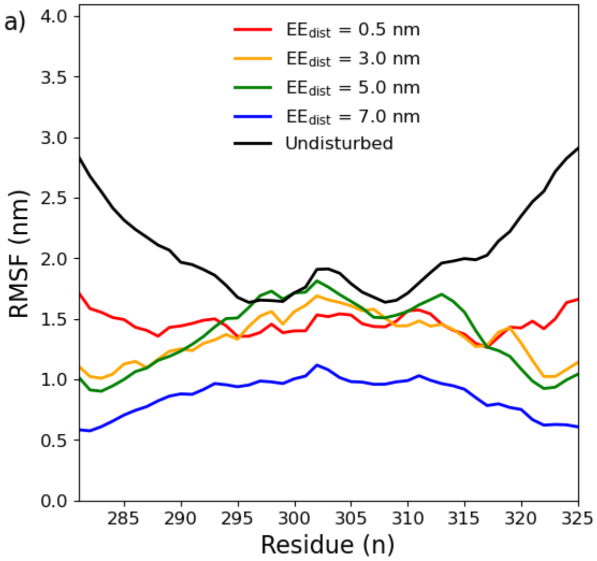

Figure S9: Root-mean-square fluctuation (RMSF) displaying the conformational flexibility of the unrestrained (black) and restrained (colored) trajectories at different end-to-end distances (EE_{dist}).

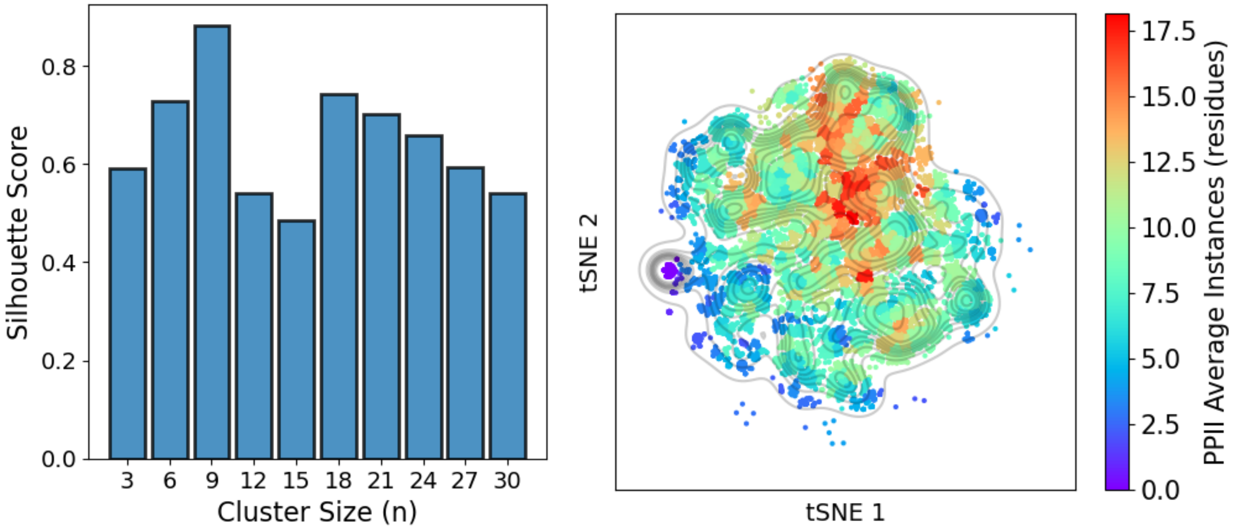

Figure S10: Silhouette scores (left) generated at different cluster sizes for the clustering of conformations based on the tSNE dimensionally reduced surface with individual values displayed (right).
